# Neuropeptide visualization using split GFP in live *C. elegans*

**DOI:** 10.1371/journal.pone.0355191

**Published:** 2026-08-19

**Authors:** Binta Maria Aleogho, Rikuou Yokosawa, Kentaro Noma

**Affiliations:** 1 Group of Nutritional Neuroscience, Neuroscience Institute, Graduate School of Science, Nagoya University, Nagoya, Japan; 2 Group of Microbial Motility, Department of Biological Science, Division of Natural Science, Graduate School of Science, Nagoya University, Nagoya, Japan; NCSU CVM: NC State University College of Veterinary Medicine, UNITED STATES OF AMERICA

## Abstract

Neuropeptides play essential roles as signaling molecules in the nervous system of animals. Visualization of neuropeptides in experimental settings has advanced through two main approaches: using antibody-based methods and fluorescent protein tagging. These conventional approaches have inherent drawbacks, such as poor antibody specificity and potential functional perturbations due to fusion with bulky, full-length fluorescent proteins. The split GFP system is a versatile tool with broad applications, yet it has been underutilized for neuropeptide labeling. We demonstrate the utility of the split GFP system in the *in vivo* visualization of two neuropeptides, INS-1 and NLP-40, released from and targeted to neuronal and non-neuronal cells and tissues in *C. elegans*. Released neuropeptides were successfully visualized by inserting the 16-amino acid GFP11 tag to the sequence of neuropeptides of interest and complementing it with extracellular GFP1–10 fused to a membrane anchor protein, CD4. We show that fusion with the GFP11 tag does not perturb INS-1 function. Furthermore, we explore the *in vivo* regulation of neuropeptide release using genetic approaches. Our findings uncover a new application of the split GFP system which offers advantages over conventional methods for labeling neuropeptides and new insights into the regulation of neuropeptide secretion.

## Introduction

Neuropeptides are short amino-acid sequences that modulate synaptic activity and function as both local neurotransmitters and long-range signaling molecules [1–4]. Initially synthesized in an inactive form, they undergo enzymatic processing within the endoplasmic reticulum, Golgi apparatus, and large dense core vesicles (DCVs) [5] before being released from neurons or non-neuronal tissues, such as the intestine in *C. elegans* [6–8]. Once released, they activate cell-surface receptors to regulate various metabolic, cellular, and physiological functions including growth, development, learning, memory, locomotion, egg laying, dauer formation, immunity, longevity, and social behavior [9–15]. *C. elegans* harbors at least 154 neuropeptide precursor genes, which encode over 300 distinct neuropeptides [16,17], classified into insulin (INS)-like peptides (ILPs), FMRFamide (Phe-Met-Arg-Phe-NH2)-related peptides (FLPs), and neuropeptide-like proteins (NLPs), which are non-insulin, non-FMRFamide-related peptides [18]. Recently, we have shown that neuropeptides influence age-dependent neuronal hyperactivity [19] and may mediate intestine-to-neuron signaling in response to aging and diet in a *C. elegans* behavior [20]. Given their critical roles in diverse biological processes, further advancements in neuropeptide visualization tools are essential for precisely monitoring their release, distribution, and functional mechanisms.

Existing methods for detecting neuropeptides have notable drawbacks. Immunochemistry using targeted antibodies can identify neuropeptide families [21] but requires sample fixation, which prevents live imaging and alters cellular morphology and molecular interactions. Additionally, developing specific antibodies is expensive and difficult due to structural similarities among neuropeptides [22,23], leading to cross-reactivity issues [8,21,24]. Fluorescent protein tagging has provided an alternative approach, enabling real-time visualization of neuropeptides in live animals [25]. However, fluorescent tags consisting of hundreds of amino acids, are considerably larger than neuropeptides, which are typically only tens of amino acids long. Moreover, labeling with these tags makes it challenging to distinguish intracellular neuropeptides in secreting cells from those that have been secreted. These limitations highlight the need for more precise tools to study neuropeptide release dynamics.

Split fluorescent proteins offer a refined method for studying molecular interactions and protein localization in living cells [26]. A common example of this technique is the split GFP system, consisting of two fragments—GFP1–10 and GFP11—that do not fluoresce by themselves, but can spontaneously and irreversibly reassemble to form a functional fluorescent GFP molecule when brought into close proximity with each other, such as being colocalized within the same cellular compartment [26,27]. GFP11, being the smaller of the two fragments, is typically fused to the protein of interest, which minimizes disruption to protein solubility and expression [26]. This technique has been widely applied across various organisms to map intracellular [28], ER-associated, and viral protein topologies [29], as well as for labeling and quantifying cell-surface proteins [30] and organelle contact sites [31]. In *C. elegans*, split GFP, split Cherry2, and split wrmScarlet have been utilized to identify neuronal synapses [32,33], visualize ribosomes in a tissue-specific manner [34], and label endogenous proteins [35,36].

Despite its plethora of applications, split fluorescent proteins have been underutilized for neuropeptide research. Here, we adapted the split GFP system to visualize two neuropeptides in live *C. elegans*. We detect INS-1 and NLP-40 by the assembly of GFP from the self-complementation of GFP1–10 and GFP11 expressed on different cells and tissues. Furthermore, we confirm the effectiveness of the split GFP system *in vivo* by demonstrating that it can report long-range neuropeptide dispersion and feeding-state-dependent dynamics. We propose that the split GFP-based approach is powerful for studying neuropeptide detection with greater specificity and minimal cellular disruption.

## Results

### Strategy for visualizing neuropeptide release using split GFP

**Fig 1:**
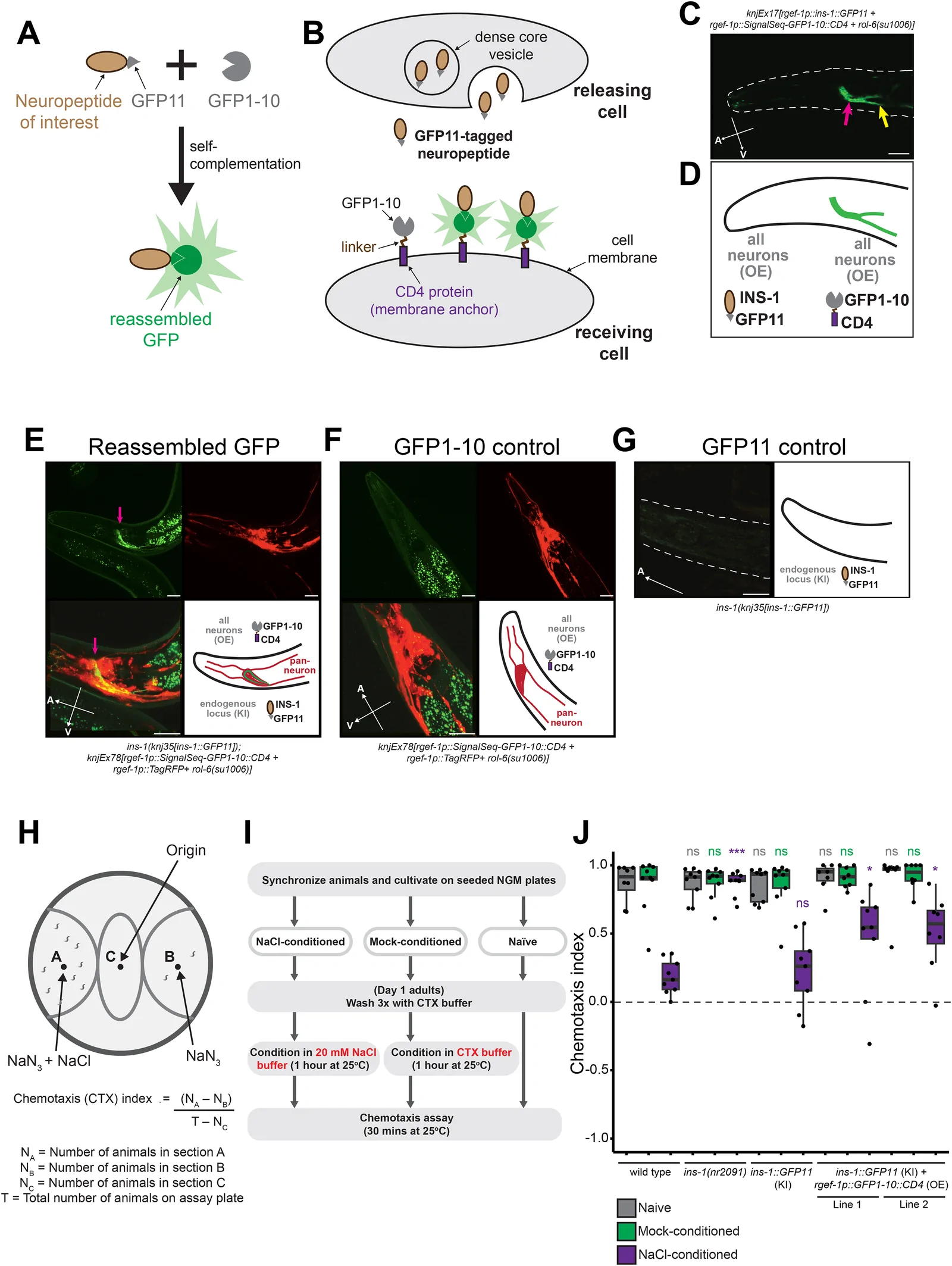
Split GFP labels INS-1 without interfering with its function. **(A and B)** Schematics of neuropeptide labeling using split GFP. **(A)** Schematic of split GFP self-complementation. The neuropeptide of interest is tagged with GFP11. **(B)** GFP11-tagged neuropeptide exocytosed from the dense core vesicles in the releasing cell binds to GFP1–10, which is fused by a linker to a membrane anchor CD4 protein on the receiving cell. **(C and D)** Split GFP labels INS-1. **(C)** Representative confocal image of the head of an animal expressing both *ins-1*::GFP11 and GFP1–10::CD4 pan-neuronally, as shown in the indicated genotype. INS-1 fluorescence from the reassembled GFP was seen in the nerve ring (magenta arrow) and ventral nerve cord (yellow arrow). White dashed lines represent the animal’s border. White arrows represent the animal’s orientation. A: anterior; V: ventral. Scale bar is 25 µm. **(D)** Schematic depicts the identities of INS-1-releasing and receiving cells in **(C)**, and the split GFP parts expressed. OE: overexpression. **(E–G)** Split GFP labels endogenous INS-1. **(E)** Representative confocal image of the head of an animal expressing *ins-1*::GFP11 endogenously and GFP1–10::CD4 pan-neuronally, as shown in the indicated genotype. Endogenous INS-1 fluorescence from the reassembled GFP was seen in the nerve ring (magenta arrow). **(F and G)** Representative confocal images of both negative control strains derived from outcrossing the strain in **(E)**. Each outcrossed strain expresses either of both split GFP parts as shown in the indicated genotypes. No green fluorescence was observed in either strain except autofluorescence in the intestine in **(F)**. Confocal images displayed as quadrants represent the green (top left), red (top right), and merge (bottom left) channels. Images in the merge channels have been magnified. Schematics (bottom right) depict the identities of endogenous INS-1-releasing and receiving cells (or tissues), and which of the split GFP parts are expressed. OE: overexpression; KI: knock-in. White dashed lines represent the animal’s border. White arrows represent the animal’s orientation. A: anterior; V: ventral. Scale bars are 25 µm. (H) Schematic of a salt chemotaxis assay plate. A salt gradient was generated on one side (Point A) of the plate, and animals were spotted at the origin (Point C) and allowed to roam freely. Chemotaxis (CTX) index was calculated using the indicated formula. (I) Schematic showing the workflow of the salt chemotaxis assay. (J) Box and whisker plots of chemotaxis indices of wild type, *ins-1(nr2091)* mutants, *ins-1*::GFP11 KI, and *ins-1*::GFP11 KI animals co-expressing GFP1–10::CD4 (n = 7–9 assays). Statistical analysis was done using the Kruskal Wallis test followed by a post-hoc Steel test for corresponding comparisons with control within a condition, as represented by the distinct asterisk colors. *p ≤ 0.05, ***p ≤ 0.001, and ‘ns’ indicates not significant (p > 0.05).

Our strategy for neuropeptide visualization using the split GFP tool was to fuse the GFP11 tag to candidate neuropeptides and allow self-complementation with GFP1–10 expressed on the extracellular membranes of target cells (Fig 1A and 1B). The 16 amino-acid GFP11 tag is much smaller than the full-length GFP tag, which consists of 238 amino acids. Due to this significant difference in size, we reasoned that using GFP11 as a tag would reduce the likelihood of interference with neuropeptide function and endogenous localization. Hence, we generated transgenic strains expressing candidate neuropeptides fused with GFP11 at their C-termini. We also expressed complementary strains expressing N-terminal GFP1–10 fusions of the human T-cell protein, CD4 (Fig 1B) [32]. In this design, CD4 protein functions as a membrane anchor since its natural extracellular ligand, the MHC class II protein, is not endogenously expressed in *C. elegans* [32]*.* Since our initial focus was on labeling neuropeptide release rather than their interaction with target cells, using the CD4 protein as a membrane anchor, as in the previous report [32], was a first step to assess the feasibility of the split GFP tool before considerations for the endogenously expressed, cognate receptors of the candidate neuropeptides. Thus, the GFP1–10::CD4 construct was designed to display GFP1–10 on the exterior of the neuropeptide-receiving cell membrane (Fig 1B). Since the two split GFP fragments are non-fluorescent on their own, we reasoned that the appearance of green fluorescence would indicate GFP reassembly triggered by neuropeptide release.

### Split GFP visualizes INS-1 *in vivo*

As a proof of concept, we tested INS-1 as a first candidate neuropeptide. Insulin-like peptides and insulin signaling are required for various *C. elegans* physiological processes, including development, aging, dauer formation, learning and memory, longevity, and fat accumulation [37,38]. Structurally, INS-1 is one of the most similar peptides to human insulin among all the *C. elegans* INS peptides, as it possesses a C-peptide between the B and A chains [39]. Furthermore, INS-1 is a key neuropeptide in the integration of behavior with the functional state of the animal during learning paradigms such as thermotaxis [40] and salt chemotaxis [41].

First, we generated a strain overexpressing (OE) *ins-1*::GFP11 and GFP1–10::CD4, both driven by the pan-neuronal *rgef-1* promoter. In these animals, we observed strong green fluorescence in the nerve ring—a circular bundle of neurites surrounding the pharynx and containing a dense network of synapses—indicating GFP reassembly (Fig 1C **and** 1D). Weaker fluorescence was also detected in the somas of a subset of head neurons. The pronounced signal in the nerve ring region may reflect the ubiquitous activity of the *rgef-1* promoter and the accumulation of GFP1–10::CD4 in this area.

Next, we asked whether the reassembled GFP observed using overexpression from extrachromosomal arrays of INS-1 could be replicated with endogenously expressed INS-1. To this end, we generated a knock-in (KI) strain of *ins-1*::GFP11 with pan-neuronal overexpression of GFP1–10::CD4. Reassembled GFP signals in the nerve ring were similarly observed in these animals (Fig 1E). As negative controls, we outcrossed this strain to isolate animals expressing only GFP1–10::CD4 or *ins-1*::GFP11. As expected, neither group of control animals exhibited green fluorescence independently, other than the intestinal autofluorescence (Fig 1F and 1G). These results indicate that the split GFP tool can visualize endogenous INS-1. Because the same neurons might express both GFP1–10::CD4 and *ins-1*::GFP11 in both configurations in Fig 1C–1E, we cannot exclude the possibility that the reconstituted GFP signals might originate from intracellular compartments such as ER, Golgi, and endosomes in addition to potential extracellular interactions.

Since the binding between INS-1::GFP11 and GFP1–10::CD4 is thought to be irreversible, the observed signals likely reflect the accumulation of past secretion events. To assess whether split GFP signal accumulates over time, we quantified fluorescence intensity of *ins-1*::GFP11 KI and pan-neuronal GFP1–10::CD4 at different adult ages (**S1A–S1D Fig**). GFP signal increased progressively from Day 1 to Day 5 (**S1E Fig**), consistent with integration of reconstituted GFP signals.

As with any protein labeling method, validating the functionality of the labeled protein is essential. To assess the functionality of INS-1 in the *ins-1*::GFP11 KI animals, we performed salt chemotaxis assays (Fig 1H–1J). Under normal laboratory conditions, *C. elegans* is attracted to salts like NaCl when fed but displays an aversive behavior when starved in the presence of NaCl. *ins-1(nr2091)* mutants, however, are defective in this aversive learning behavior [41]. Animals were divided into three groups: NaCl-conditioned, Mock-conditioned (without NaCl), and Naïve (Fig 1I). The defect observed in *ins-1(nr2091)* mutants in the previous study was successfully recapitulated (Fig 1J; [41]). The *ins-1*::GFP11 KI animals behaved similarly to the wild type across all three groups (Fig 1J), indicating normal function of GFP11-tagged INS-1. Moreover, co-expression of GFP1–10::CD4 with *ins-1*::GFP11 did not significantly alter NaCl chemotaxis in the Naïve or Mock-conditioned groups and only slightly prevented learning-induced changes in chemotaxis index following NaCl-conditioning (Fig 1J).

### Split GFP visualizes INS-1 released from specific cells and tissues

Building on the finding that GFP11 tagging does not interfere with INS-1 function, we investigated whether the split GFP tool could label INS-1 released from specific cells and tissues. Moreover, we wanted to prove that GFP11-tagged neuropeptide and GFP1–10::CD4 can reconstitute extracellularly to show fluorescence. To this end, neuropeptide visualization strains were generated from a genetic cross between two independent strains, each carrying one of the split GFP transgenes driven by a cell- or tissue-specific promoter. Rather than generating a strain that expresses both split GFP transgenes in single extrachromosomal arrays, generating strains in this manner would prevent possible crosstalk between promoters and enable precise determination of the origin of the observed green fluorescence.

**Fig 2:**
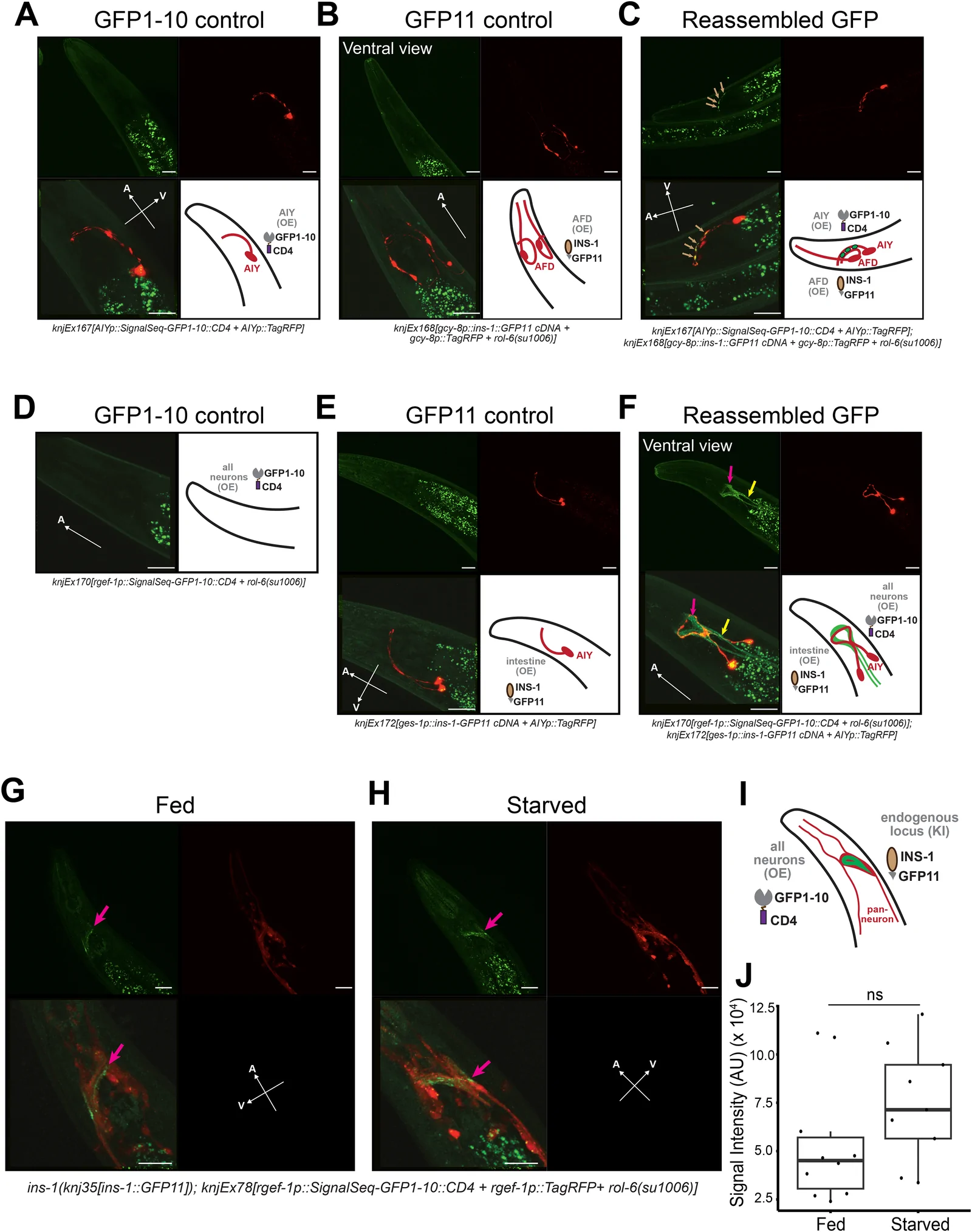
Split GFP labels INS-1 released from specific neurons and tissues. **(A–H)** Confocal images displayed as quadrants represent the green (top left), red (top right), and merge (bottom left) channels. Images in the merge channels have been magnified. Schematics (bottom right) depict the identities of INS-1-releasing and receiving cells (or tissues), and which of the split GFP parts they express. OE: overexpression; KI: knock-in. White arrows represent the animal’s orientation. A: anterior; V: ventral. Scale bars are 25 µm. **(A–C)** Split GFP labels INS-1 at AFD-AIY synapses. Representative confocal images of the head of an animal expressing GFP1–10::CD4 in AIY **(A)**, *ins-1*::GFP11 in AFD **(B)**, or both split GFP parts **(C)**, as shown in the indicated genotypes. **(C)** INS-1 fluorescence from the reassembled GFP was seen in the AFD-AIY synaptic region (light brown arrows). **(D–F)** Split GFP labels INS-1 released from the intestine. Representative confocal images of the head of an animal expressing GFP1–10::CD4 pan-neuronally **(D)**, *ins-1*::GFP11 in the intestine **(E)**, or both split GFP parts **(F)**, as shown in the indicated genotypes. **(F)** INS-1 fluorescence from the reassembled GFP was seen in the nerve ring (magenta arrow) and ventral nerve cord (yellow arrow). **(G and H)** Representative confocal images of the head of fed and starved animals expressing *ins-1*::GFP11 endogenously and GFP1–10::CD4 pan-neuronally, as shown in the indicated genotype. Starvation induced increased, but not significantly different, INS-1 signal in the nerve ring of animals (magenta arrow). (I) Schematic depicts the identities of INS-1-releasing and receiving cells (and tissues) in **(G and H)**, and the split GFP parts they express. (J) Quantification of reassembled GFP signal in the nerve ring of animals in **(G and H)** (n = 10, 9 animals). Statistical analysis was done using the Mann Whitney U/Wilcoxon rank sum exact test. ‘ns’ indicates not significant (p > 0.05).

We utilized a pair of neurons, AFD and AIY because AFD sensory neurons are known to synapse onto AIY interneurons in the *C. elegans* thermotaxis circuit [42,43]. Although AFD does not endogenously express *ins-1* [44], AFD and AIY neuron promoters are highly specific and non-overlapping, allowing us to exclude the possibility of intracellular GFP assembly within the same cell. We generated a strain overexpressing GFP1–10::CD4 in AIY neurons (Fig 2A) and another strain overexpressing *ins-1*::GFP11 in AFD neurons (Fig 2B). Neither strain had green fluorescence. On the other hand, animals from the genetic cross of these two strains displayed green punctate signals along the nerve ring, where AFD and AIY contact (Fig 2C), demonstrating that split GFP can visualize neuropeptide released between synaptic partners.

*ins-1* is expressed in the intestine, muscles, and neurons of *C. elegans* [6,38]. To examine whether the split GFP can report long-range signaling among distant, non-synaptic partners, we generated two independent extrachromosomal transgenic strains: one overexpressing GFP1–10::CD4 under the control of the pan-neuronal *rgef-1* promoter (Fig 2D), and the other overexpressing *ins-1*::GFP11 under the control of the intestine-specific *ges-1* promoter (Fig 2E). Animals from a genetic cross of these two strains exhibited strong, dispersed green signals in the nerve ring and ventral nerve cord (Fig 2F). This finding demonstrates that INS-1::GFP11 released from the intestine traveled to the nerve ring region and that long-range neuropeptide dispersion can be visualized with split GFP.

*ins-1* modulates starvation-dependent behavioral plasticity, including thermotaxis [40] and salt chemotaxis [41]. Thus, we investigated whether the split GFP system could report INS-1 secretion under different feeding conditions. Using the *ins-1*::GFP11 KI animals overexpressing GFP1–10::CD4 pan-neuronally (Fig 1E), we examined reassembled GFP signals in continuously fed animals and in those starved for 3 hours by transferring to plates without food prior to observation. Compared to well-fed animals, animals starved for 3 hours exhibited a trend of increase in reconstituted GFP signal along the nerve ring, while we did not see any consistent changes in the localization patterns (Fig 2G–2J). Although, the increase in signal was not statistically significant, this result supports previous reports that feeding state differentially regulates insulin peptides’ release in *C. elegans* [38,39] and demonstrates the potential utility of the split GFP system to report neuropeptide secretion dynamics.

Additionally, we assessed secretion of INS-1 by coelomocyte uptake using intestine-derived INS-1 tagged with a full-length GFP (S2A–S2C Fig). We quantified GFP fluorescence in anterior, midbody, and posterior coelomocytes in fed animals and those starved for 3 hours. In contrast to the split GFP labeling, starvation caused a significant reduction in INS-1::GFP accumulation in coelomocytes, despite comparable intestinal fluorescence (S2D Fig).

### Split GFP visualizes NLP-40

**Fig 3:**
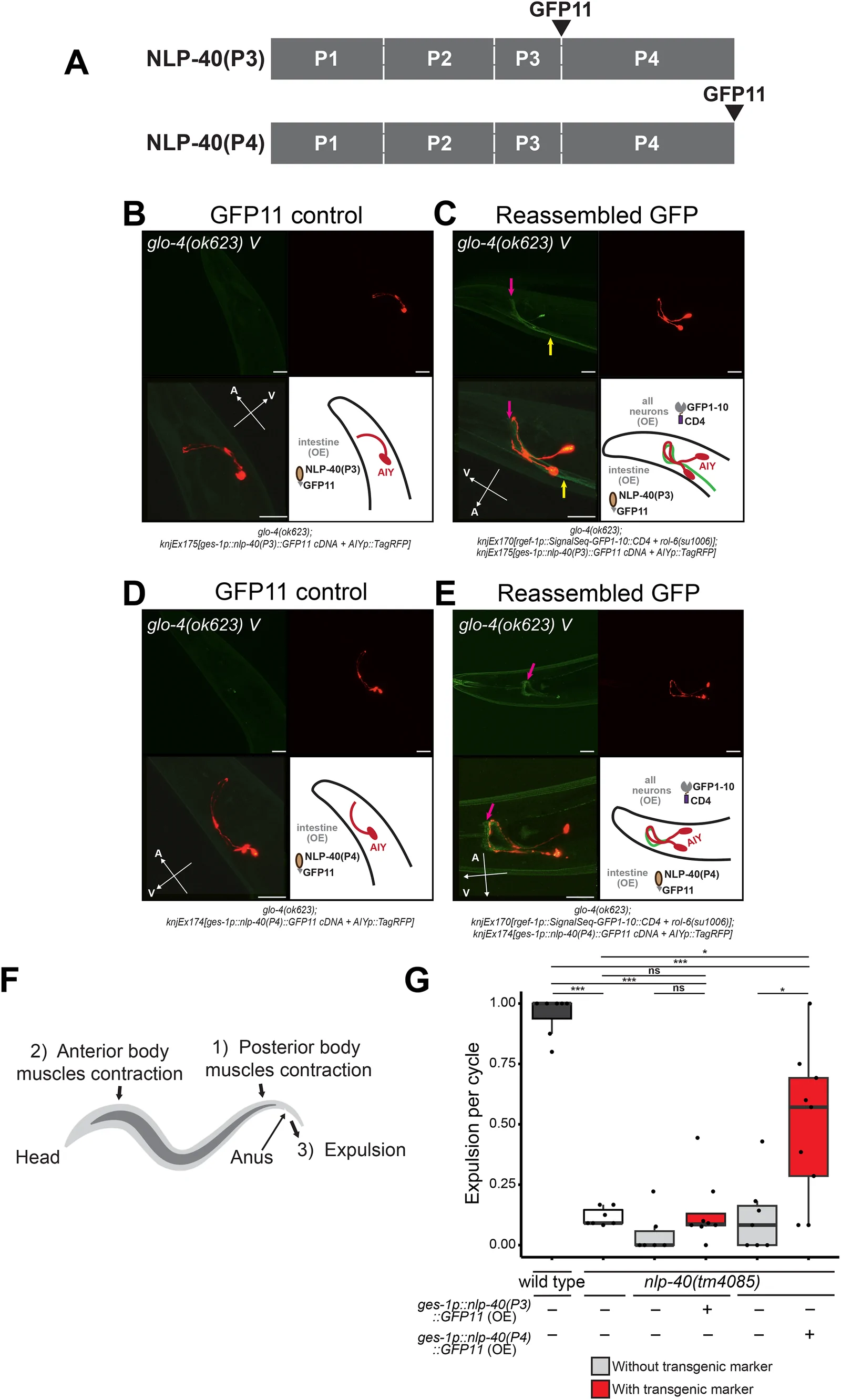
Split GFP labels NLP-40 released from the intestine. **(A)** Schematic showing the design of the two NLP-40 constructs used. Black arrowheads represent the position of the GFP11 tag in each construct. **(B–E)** Confocal images displayed as quadrants represent the green (top left), red (top right), and merge (bottom left) channels. Images in the merge channels have been magnified. Schematics (bottom right) depict the identities of NLP-40-releasing and receiving cells or tissues, and which of the split GFP parts they express. OE: overexpression. White arrows represent the animal’s orientation. A: anterior; V: ventral. Scale bars are 25 µm. **(B–C)** Split GFP labels NLP-40(P3) released from the intestine. Representative confocal images of the head of an animal expressing *nlp-40(P3)*::GFP11 in the intestine **(B)**, or both split GFP parts **(C)**, as shown in the indicated genotypes. **(C)** NLP-40(P3) fluorescence from the reassembled GFP was seen in the nerve ring (magenta arrow) and ventral nerve cord (yellow arrow). **(D–E)** Split GFP labels NLP-40(P4) released from the intestine. Representative confocal images of the head of an animal expressing *nlp-40(P4)*::GFP11 in the intestine **(D)**, or both split GFP parts **(E)**, as shown in the indicated genotypes. **(E)** NLP-40(P4) fluorescence from the reassembled GFP was seen in the nerve ring (magenta arrow). (F) Schematic of an animal showing the sequence of events during defecation. Thick black arrows indicate the site on the animal at which each event takes place. (G) Box and whisker plots of expulsion per cycle for wild type and *nlp-40(tm4085)* mutants (n = 6–8 assays). The mutants express either the *ges-1p::nlp-40(P3)::GFP11* (OE) or *ges-1p::nlp-40(P4)::GFP11* (OE) transgene (+) or not (–). Statistical analysis was done using the Mann Whitney U/Wilcoxon rank sum exact test. *p ≤ 0.05, ***p ≤ 0.001 and ‘ns’ indicates not significant (p > 0.05).

To address the versatility of split GFP labeling, we examined a neuropeptide in a different class, NLP-40. NLP-40 is expressed in the intestine and regulates *C. elegans* defecation [45] and axon regeneration [46]. It consists of four predicted mature peptides—P1 to P4—with the P3 peptide reported to be necessary and sufficient for NLP-40 function in defecation [45]. To visualize this neuropeptide, we designed two types of NLP-40::GFP11 constructs. In one construct, GFP11 was fused to the C-terminus of the P3 peptide (*nlp-40*(P3)::GFP11) and in the other, to the C-terminus of the P4 peptide (*nlp-40*(P4)::GFP11) (Fig 3A). We created these constructs to identify potential differences in the expression and dynamics between an internal P3 peptide versus a terminal P4 peptide.

Transgenic strains overexpressing NLP-40 (P3 or P4) in the intestine were generated (Fig 3B–3E) from crosses with the strain overexpressing GFP1–10::CD4 pan-neuronally (Fig 2D). Animals expressing either *nlp-40*(P3)::GFP11 or *nlp-40*(P4)::GFP11 alone did not show any green fluorescent signals (Fig 3B **and** 3D). To visualize any potential intestinal signals, these crosses were performed in a *glo-4(ok623)* mutant background (Fig 3B and 3D), which shows a defect in gut granule biogenesis [47] and lacks the natural autofluorescence observed in the wild type. No intestinal signals were observed in the strains resulting from these crosses, suggesting no intracellular reconstitution. On the other hand, reassembled GFP signals were observed in the nerve rings and ventral nerve cords of animals expressing NLP-40 (P3 or P4) intestinally together with GFP1–10::CD4 pan-neuronally (Fig 3C **and** 3E).

*C. elegans* undergoes a rhythmic defecation motor program lasting approximately 50 seconds and characterized by three sequential events: contraction of posterior body muscles, contraction of anterior body muscles, and finally, contraction of anal muscles, which results in the expulsion of intestinal waste contents (Fig 3F; [48,49]). *nlp-40* mutants exhibit defects in the expulsion step (Fig 3G; [45]). To confirm the normal function of NLP-40 after tagging with GFP11, we performed genetic crosses and assessed whether strains expressing intestinal *nlp-40*(P3)::GFP11 or *nlp-40*(P4)::GFP11 could rescue defecation defects of *nlp-40(tm4085)* mutants. Although, there was no rescue of the defecation phenotype in *nlp-40(tm4085)* mutants carrying the *nlp-40*(P3)::GFP11 transgene, those carrying the *nlp-40*(P4)::GFP11 transgene displayed an expulsion phenotype intermediate to those of the wild type and *nlp-40(tm4085)* mutants (Fig 3G). We note that the *nlp-40*(P4)::GFP11 strain should express untagged *nlp-40*(P3) (Fig 3A). These results indicate that GFP11 likely interfered with the function and/or proper cleavage of the rescuing P3 peptide and support the results from the previous study showing the necessity and sufficiency of the P3 peptide in *C. elegans* defecation [45]. As negative controls, we also evaluated the non-transgenic siblings and observed no rescue (Fig 3G).

### Visualizing neuropeptides in mutants of neuropeptide processing and release

To determine whether the split GFP system can report defects in neuropeptide release, we analyzed three mutants with impairments in neuropeptide precursor processing or neuropeptide release mechanisms: *unc-31(e928)* mutants, which are defective in the *C. elegans* homolog of the mammalian CAPS protein (Ca^2+^-dependent activator protein for secretion), a key factor in docking, priming and exocytosis of DCVs during neuropeptide secretion [50–52]; *snt-2(tm1711)* mutants, which are defective in synaptotagmin, a principal calcium sensor involved in the exocytosis of DCVs and synaptic vesicles (SVs) [53,54]; and *egl-21(n476)* mutants, which are defective in carboxypeptidase E, an enzyme required for the cleavage and removal of C-terminal basic residues during neuropeptide processing [55]. We expected that these mutants, owing to their specific defects in neuropeptide processing or release, would show little to no reassembled GFP signals when assessed using the split GFP system.

**Fig 4:**
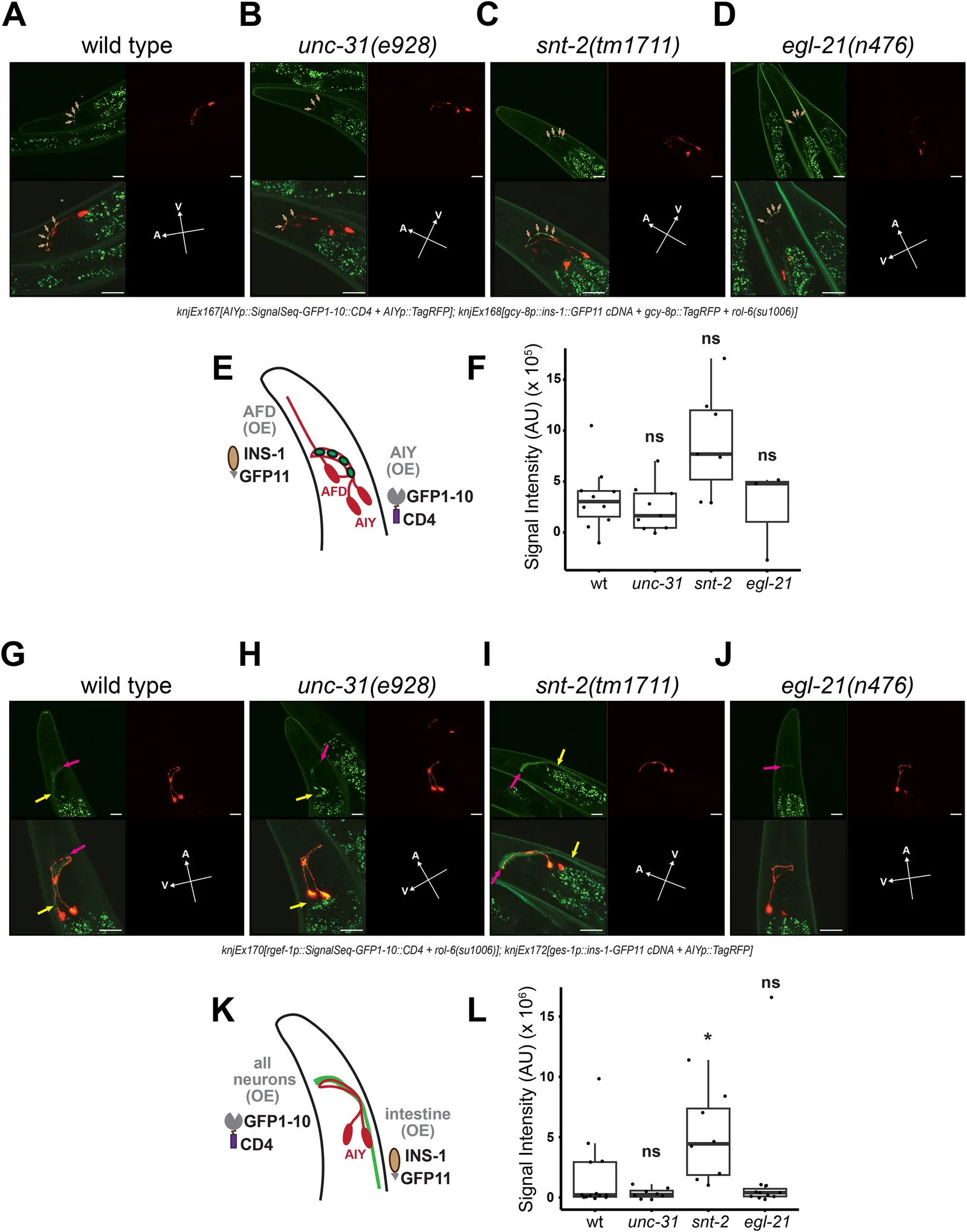
Mutants of neuropeptide processing and release exhibit largely unaffected or increased INS-1 signals. Confocal images displayed as quadrants representing the green (top left), red (top right), and merge (bottom left) channels. Images in the merge channels have been magnified. White arrows (bottom right) represent the animal’s orientation. A: anterior; V: ventral. Scale bars are 25 µm. **(A–D)** Representative confocal images of the head of wild type and mutant animals expressing GFP1–10::CD4 in AIY and *ins-1*::GFP11 in AFD, as shown in the indicated genotype. INS-1 fluorescence from the reassembled GFP was seen in the AFD-AIY synaptic region (light brown arrows). Images in **(A)** are the same as those in Fig 2C. (E) Schematic depicts the identities of INS-1-releasing and receiving cells in **(A–D)**, and the split GFP parts they express. OE: overexpression. (F) Quantification of reassembled GFP puncta in the AFD-AIY synaptic region of animals in **(A–D)** (n = 3–10 animals). Statistical analysis was done using the Kruskal Wallis test followed by a post-hoc Steel test for pairwise comparisons with wild type. ‘ns’ indicates not significant (p > 0.05). **(G–J)** Representative confocal images of the head of wild type and mutant animals expressing GFP1–10::CD4 pan-neuronally and *ins-1*::GFP11 in the intestine, as shown in the indicated genotype. INS-1 fluorescence from the reassembled GFP is seen in the nerve ring (magenta arrows) and ventral nerve cord (yellow arrows). (K) Schematic depicts the identities of INS-1-releasing and receiving cells in **(G–J)**, and the split GFP parts they express. OE: overexpression. (L) Quantification of reassembled GFP signal in the nerve ring of animals in **(G–J)** (n = 8–13 animals). Statistical analysis was done using the Kruskal Wallis test followed by a post-hoc Steel test for pairwise comparisons with wild type. *p ≤ 0.05 and ‘ns’ indicates not significant (p > 0.05).

To investigate the regulation of INS-1 release, we crossed *unc-31*, *snt-2*, and *egl-21* mutants with the transgenic strain overexpressing GFP1–10::CD4 in AIY and *ins-1*::GFP11 in AFD (Figs 2C and 4A–4D). In all mutant backgrounds, there was no statistically significant change in intensity of the synaptic green puncta, compared to the wild type although *snt-2* mutants had a trend of increased signals (Fig 4A–4F). Additionally, we crossed *unc-31*, *snt-2*, and *egl-21* mutants with the transgenic strain overexpressing *ins-1*::GFP11 in the intestine and GFP1–10::CD4 pan-neuronally (Figs 2F and 4G–4L). In *unc-31* and *egl-21* mutant backgrounds, there was no change in the signal intensities of the nerve ring and ventral nerve cord relative to the wild type (Fig 4H, 4J, and 4L). In contrast, the *snt-2* mutant background showed an increase in signal intensity in the nerve ring and ventral nerve cord (Fig 4I and 4L). These results suggest that *unc-31* and *egl-21* have negligible effects on the regulation of INS-1 release and processing, respectively, and that *snt-2* negatively regulates INS-1 release from the intestine.

**Fig 5:**
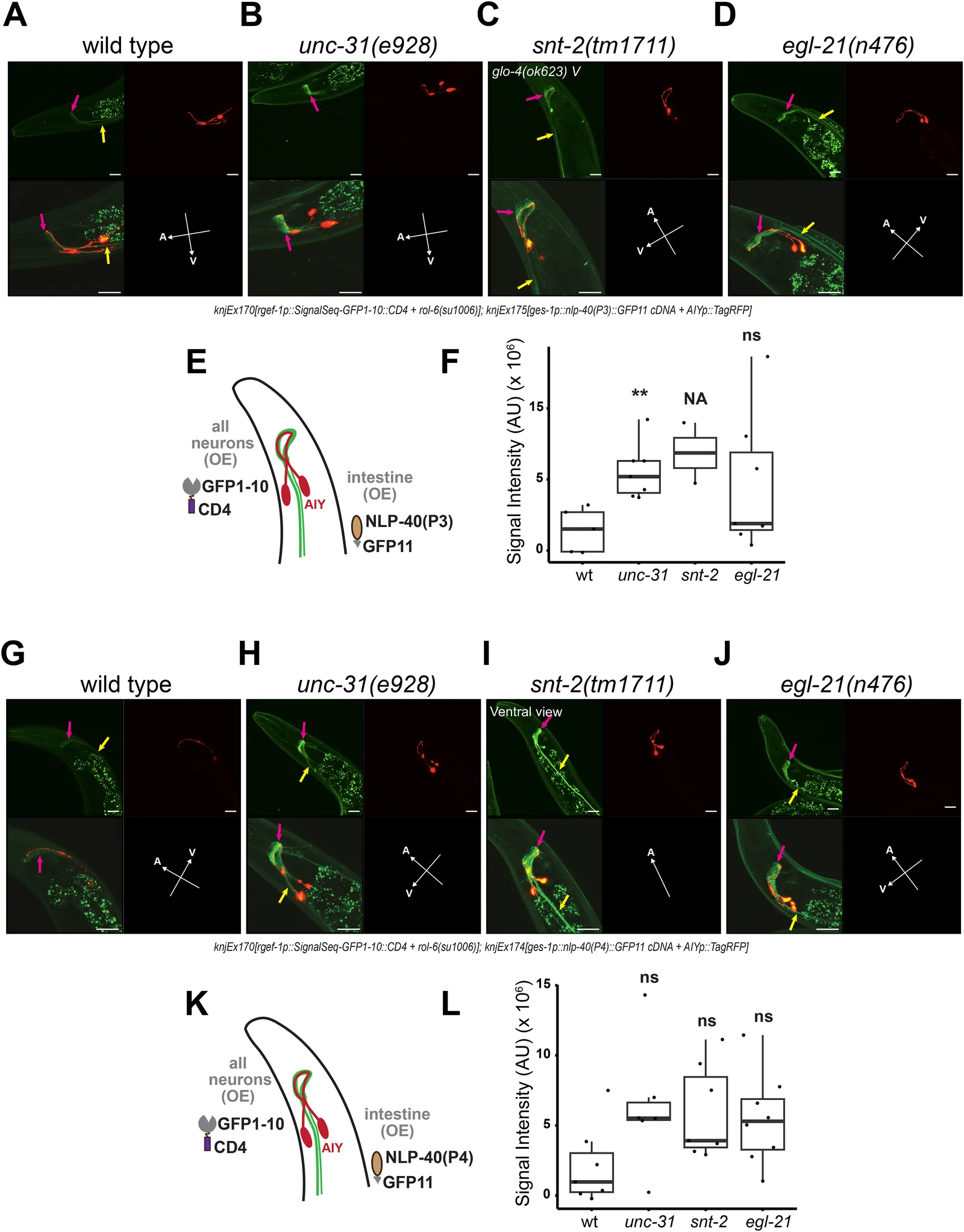
Mutants of neuropeptide processing and release exhibit largely unaffected or increased NLP-40 signals. Confocal images displayed as quadrants representing the green (top left), red (top right), and merge (bottom left) channels. Images in the merge channels have been magnified. White arrows (bottom right) represent the animal’s orientation. A: anterior; V: ventral. Scale bars are 25 µm. **(A–D)** Representative confocal images of the head of wild type and mutant animals expressing GFP1–10::CD4 pan-neuronally and *nlp-40(P3)*::GFP11 in the intestine, as shown in the indicated genotype. NLP-40(P3) fluorescence from the reassembled GFP was seen in the nerve ring (magenta arrows) and ventral nerve cord (yellow arrows) of animals. (E) Schematic depicts the identities of NLP-40(P3)-releasing and receiving cells in **(A–D)**, and the split GFP parts they express. OE: overexpression. (F) Quantification of reassembled GFP signal in the nerve ring of animals in **(A–D)** (n = 2–7 animals). Statistical analysis was done using the Kruskal Wallis test followed by a post-hoc Steel test for pairwise comparisons with wild type. **p ≤ 0.01 and ‘ns’ indicates not significant (p > 0.05). ‘NA’ indicates not applicable as *snt-2* was not included in the statistical analysis due to the presence of the *glo-4* background. **(G–J)** Representative confocal images of the head of wild type and mutant animals expressing GFP1–10::CD4 pan-neuronally and *nlp-40(P4)*::GFP11 in the intestine, as shown in the indicated genotype. NLP-40(P4) fluorescence from the reassembled GFP is seen in the nerve ring (magenta arrows) and ventral nerve cord (yellow arrows). (K) Schematic depicts the identities of INS-1-releasing and receiving cells in **(G–J)**, and the split GFP parts they express. OE: overexpression. (L) Quantification of reassembled GFP signal in the nerve ring of animals in **(G–J)** (n = 6–8). Statistical analysis was done using the Kruskal Wallis test followed by a post-hoc Steel test for pairwise comparisons with wild type. ‘ns’ indicates not significant (p > 0.05).

To investigate the regulation of NLP-40 using the split GFP system, we crossed *unc-31*, *snt-2*, and *egl-21* mutants with strains overexpressing *nlp*-40::GFP11 (P3 or P4 peptide-tagged) intestinally and GFP1–10::CD4 pan-neuronally (Figs 3C and 3E and 5A–5L). In all mutants tested, the intensity of the reassembled GFP signal in the nerve ring tended to increase compared to the wild type (Fig 5A–5L) but was only statistically significant in *unc-31* mutants expressing *nlp-40*(P3)::GFP11 (Fig 5B and 5F). In Fig 5C, the strain having the wild-type background could not be isolated, hence, images in the *glo-4(ok623)* mutant background are shown and excluded from the statistical analysis. Similar to INS-1, NLP-40 release was not downregulated in these mutants. Rather, the increased signals suggest that NLP-40 processing and release are negatively regulated by *unc-31*, *snt-2*, and *egl-21*. Furthermore, we independently assessed secretion by coelomocyte uptake using an intestine-derived NLP-40 tagged with full-length GFP (S3A–S3E Fig). We quantified GFP fluorescence in anterior, midbody, and posterior coelomocytes in wild-type*, unc-31*, *snt-2,* and *egl-21* animals. Coelomocyte signal was not statistically different among the four genotypes (S3F Fig), indicating that overall intestinal expression and bulk extracellular release of NLP-40::GFP are preserved in these mutants.

## Discussion

This study proposes a novel application of the split GFP system for neuropeptide labeling in *C. elegans*. By tagging two candidate neuropeptides—INS-1 and NLP-40—with a small GFP11 tag, we demonstrate their visualization from various cells and tissues in live animals. Using the split GFP tool, INS-1 was visualized at AFD-AIY synapses (Fig 2C), and INS-1 and NLP-40 released from the intestine were visualized in the *C. elegans* nerve ring (Figs 2F, 3C, and 3E). Furthermore, the efficacy of the split GFP tool was demonstrated by its ability to not interfere with the salt chemotaxis function of INS-1 (Fig 1J), label endogenously expressed INS-1 (Fig 1E), and to some degree, report the nutrient state-dependent release dynamics (Fig 2G–2J, S2 Fig, [40]), highlighting its potential for use in complex biological systems.

Distinguishing changes in neuropeptide expression from changes in release is critical when interpreting nutritional effects. Previous work suggests that *ins-1* expression is not increased during starvation [56]. Our coelomocyte uptake assay showing that coelomocyte fluorescence is significantly lower in starved animals than in fed controls (S2 Fig), indicates that starvation does not increase bulk extracellular INS-1 availability. In contrast, neuronal split GFP reconstitution signals showed a trend toward increased localization in starved conditions (Fig 2J). These results suggest that starvation does not increase INS-1 production or overall secretion into the body cavity but instead, may shift how INS-1 is routed or delivered to specific neuronal targets under starvation conditions. Our findings for NLP-40 should also be considered in relation to prior bulk secretion assays. Wang *et al*. observed reduced coelomocyte fluorescence of NLP-40 in *snt-2* mutants [45]. However, we found no significant change in coelomocyte signal across *snt-2* and other mutants (S3 Fig). While this difference may reflect assay conditions or promoter-specific expression, elevated fluorescence in the split GFP configuration (Fig 4G–4L) may reflect increased local peptide availability near the nerve ring, rather than increased global secretion. Together, these results emphasize that split GFP–based proximity labeling and coelomocyte uptake provide complementary but distinct readouts of neuropeptide trafficking and secretion.

Although the tool proved effective for detecting neuropeptide signals, analyses of neuropeptide secretion regulation using mutants from previous reports [25,45,51,57] yielded results contrary to expectations. Instead of the expected decrease of reassembled GFP signals, mutants generally exhibited minimal to no change in signal intensity and, in some cases, displayed higher signal levels than wild-type controls. There are a few possible explanations for this discrepancy. First, the secretion of candidate neuropeptides may not be positively regulated by the three regulatory genes analyzed—*unc-31*, *snt-2*, and *egl-21*—and instead, other regulatory genes may be active in the same cells. For instance, the lack of *unc-31*-dependency of the intestine-derived INS-1 and NLP-40 may be explained by weak or no expression of *unc-31* in the intestine [51]. It is possible that the secretion of neuropeptides in large non-neuronal tissues such as the intestine is less strictly dependent on canonical neuronal dense-core vesicle release machinery. Although *unc-31(e928)* is generally considered a null or strong loss-of-function mutation [51,58], the persistence of residual fluorescence signals in this study (Figs 4B and 4H; 5B and 5H) and in previous reports [25,51,52] suggests that the mutant protein retains partial activity to release neuropeptides. Regarding the *egl-21* mutant, neuropeptide processing by EGL-21 might not be required for neuropeptide secretion. Thus, testing mutants of other key neuropeptide regulatory genes and testing purely neuronally released, UNC-31–dependent neuropeptides such as FMRFamide-related peptides (e.g., NLP-21 or FLP-1) would provide an important additional benchmark.

Second, this study could not rule out the presence of reconstituted intracellular GFP signals either during synthesis or after endocytosis, and such signals may not respond to regulation as expected. Especially when both GFP1–10::CD4 and GFP11-tagged neuropeptides are expressed in the same cells, GFP complementation could occur intracellularly within the secretory pathway, rather than exclusively at the cell surface. Accordingly, experiments in which GFP fragments are broadly co-expressed (e.g., Fig 1C–1E) are interpreted as demonstration of assay feasibility and sensitivity. Nonetheless, our observations support reconstitution after the secretory pathways because GFP signals were predominantly observed along neuronal processes, which are devoid of rough ER markers [59], rather than in perinuclear regions. Especially in the experiments involving anatomically distinct source and target cells (e.g., AFD–AIY), extracellular reconstitution is strongly supported.

A limitation of this study is the reliance on extrachromosomal arrays to express GFP1–10::CD4 constructs, which can introduce mosaicism and contribute to variability. In addition, the limited amount of GFP1–10::CD4 may have become saturated, thereby hindering the detection of changes in neuropeptide secretion in the mutant backgrounds. Another limitation is that the mutant analyses relied on exogenously overexpressed INS-1 and NLP-40 using the extrachromosomal arrays, which may mask the endogenous regulation of these neuropeptides. Moreover, the method using GFP1–10::CD4 primarily reports peptide release and local dispersion near target membranes, rather than engagement to cognate receptors. Future development of knock-in–based split-GFP strategies using GFP1–10-tagged cognate receptors rather than CD4 will be important for extending this approach under fully physiological conditions to map neuropeptide signal transduction *in vivo*. In this case, the expression level of the targeted neuropeptides should be considered because split GFP system will sacrifice the signal intensity compared to the full-length GFP. Another limitation of the split GFP is the irreversible binding of GFP11 and GFP1–10, which might perturb endogenous signaling dynamics. Future optimization may address this limitation through the use of alternative split fluorescent systems with reversible complementation [60]. While additional technical optimization will be required, these extensions highlight the potential of split GFP–based strategies for dissecting neuropeptide signaling in physiologically relevant settings. Coupling this approach with behavioral, physiological, or state-dependent manipulations (e.g., starvation or circuit perturbations) would further allow peptide delivery to be linked to functional outcomes. Overall, this study provides a proof of principle to utilize split GFP in neuropeptide labeling.

## Materials and methods

### *C. elegans* maintenance and strains

All *C. elegans* strains were maintained at 23^o^C on Nematode Growth Medium (NGM) plates with *Escherichia coli* (*E. coli*, OP50) as a food source, as previously reported [61]. N2 (Bristol) was used as the wild type. Except otherwise indicated, all assays were performed on animals at Day 1 of adulthood. Strains were obtained from the Caenorhabditis Genetics Center or generated in our laboratory using standard crossing or transgenesis methods as described below [62,63]. The strains used in this study are summarized in S1 Table.

### Generation of plasmids and transgenic strains

The entry vectors, based on pCR8 (Invitrogen), and destination vectors were cloned by Gibson HiFi DNA Assembly (New England Biolabs). We inserted an 11-amino acid linker (VDGGGGSGGGG) between GFP1–10 and CD4. The entry and destination vectors were recombined using LR clonase (Invitrogen) to generate expression vectors. To generate *ins-1*::GFP11(*knj35*) knock-in animals, we used previously reported CRISPR technology [64]. Briefly, the following mixture was injected into the N2 gonad: 250 ng/µL Cas9 (IDT); 5 µM tracrRNA (IDT); 5 µM *ins-1* crRNA (KN1665); 5 µM *ins-1::GFP11* ssOligo as the repair template; 40 ng/µL pRF4 (*rol-6(su1006)*). Rol F1s were singly picked and genotyped in the next generation. The GFP11 insertion was confirmed by PCR and Sanger sequencing. To generate extrachromosomal array (overexpression) animals, plasmids were injected into the gonad of N2, *ins-1*::GFP11(*knj35*) knock-in, or *glo-4(ok623)* animals as previously reported [62]. When required, the empty vector pUC19 was added to the injection mix to bring the final plasmid concentration to ~100 ng/µL. Plasmids used in this study are summarized in S2 Table. Primer sequences, DNA templates for GFP11 knock-in, and the signal sequence for GFP1–10 targeting are summarized in S3 Table. Although *rol-6(su1006)* has been reported to influence synaptic phenotypes in male-specific neurons [65], all genotypes within each experiment used the same co-injection marker, reducing the likelihood of marker-specific effects; future work could avoid this caveat by using alternative markers or single-copy knock-in approaches.

### Fluorescence microscopy

Animals at Day 1 of adulthood were immobilized on a 4% agarose pad in M9 buffer with 5 mM levamisole. Fluorescent images were either taken by the AxioImager.A2 (Zeiss) compound microscope or the FV3000 (Olympus) and LSM880 (Zeiss) confocal microscopes. Images on the confocal microscopes were obtained with a 488-nm laser for GFP and a 561-nm laser for TagRFP. The GFP fluorescence intensity of the nerve ring, AFD-AIY synaptic puncta, or coelomocytes was quantified using ImageJ (Fiji) [66]. Displayed images are maximum-intensity projections of Z-stacks.

### Salt chemotaxis assay

NaCl chemotaxis assay was performed as previously reported [41,67]. The salt gradient was generated by excising a 5 mm-diameter agar plug from a plate containing 100 mM of NaCl, placing the plug 2 cm from the edge of the assay plate, and leaving it overnight. Shortly before the assay, the NaCl plug was removed and then, 1 µL of 0.5 M NaN_3_ was spotted on the middle of the test and control sides (see Fig 1H, Points A and B, respectively) of the plate. Fifty to 150 animals were washed off cultivation plates (Naïve) or transferred from respective conditioning buffers and placed at the center of a 9 cm-diameter assay plate (2% w/v Bacto agar, 1 mM CaCl_2_, 1 mM MgSO_4_, 5 mM K-PO_4_ (pH 6)) on which a salt gradient had been formed overnight. Animals in the “Mock-conditioned” and “NaCl-conditioned” groups were conditioned in CTX buffer (1 mM CaCl_2_, 1 mM MgSO_4_, 5 mM K-PO_4_ (pH 6)) containing 0 mM and 20 mM NaCl, respectively, before transferred to the assay plates. Animals were allowed to roam freely for 30 minutes at room temperature and were killed by applying chloroform to the lid of the assay plate. The Chemotaxis Index reflecting animals’ chemotaxis ability was calculated as shown in Fig 1H and plotted. Experiments were conducted on at least three different days to check the reproducibility of the trend, and each data point represents an independent assay plate.

### Defecation assay

The defecation assay was performed on well-fed, Day 1 adult animals similar to previous reports [45,68,69]. An animal was picked from the cultivation plate onto a new plate with OP50 and allowed to roam for about 10 minutes to recover from being picked. The animal’s posterior body muscle contractions and anal muscle contractions (the latter of which results in the expulsion of the gut waste contents) were tracked by eye under a stereomicroscope, and the timings of these contraction and expulsion events were recorded. Plots of the number of expulsion events per defecation cycle were generated.

### Statistical analysis

Box plots were generated using RStudio (https://www.r-project.org), and all data were analyzed for statistics using R (version 4.3.1). In box plots, the lower boundary, median line, and upper boundary of the boxes indicate the 25th, 50th, and 75th percentiles, while the whiskers show the minimum and maximum values. For salt chemotaxis experiments (see Fig 1J), the Kruskal Wallis test was used to analyze statistical differences, and the post-hoc Steel test for corresponding comparisons with control within a condition. For the quantification of reassembled GFP signals of fed vs starved animals (see Fig 2J) and the defecation experiment (see Fig 3G), the Mann-Whitney U/Wilcoxon rank sum exact test was used to analyze statistical differences. For, the quantification of reassembled GFP signals of mutants, (see Figs 4 and 5) the Kruskal Wallis test was used to analyze statistical differences, and the post-hoc Steel test for pairwise comparisons of each mutant with the control. For all analyses, *p ≤ 0.05, **p ≤ 0.01, and ‘ns’ indicates not significant (p > 0.05).

## Supporting information

S1 Fig Split GFP signal increases with age. **(A–C)** Representative confocal images of the head of Day 1, 3, and 5 animals expressing *ins-1*::GFP11 endogenously and GFP1–10::CD4 pan-neuronally, as shown in the indicated genotype. Animals show progressive increase in INS-1 signal in the nerve ring (magenta arrows). White arrows represent the animal’s orientation. A: anterior; V: ventral. Scale bars are 20 µm. (D) Schematic depicts the identities of INS-1-releasing and receiving cells (and tissues) in **(A–C)**, and the split GFP parts they express. (E) Quantification of reassembled GFP signal in the nerve ring of animals in **(A–C)** (n = 20, 15, 18 animals). Statistical analysis was done using the Kruskal Wallis test followed by a post-hoc Steel Dwass test for comparisons within the three age groups. Different alphabets indicate significant difference.
(PDF)

S2 Fig Starvation reduces coelomocyte uptake of INS-1::GFP. **(A and B)** Representative confocal images of the body of fed and starved animals expressing *ins-1*::GFP in the intestine, as shown in the indicated genotype. Starved animals show decreased coelomocyte uptake of INS-1::GFP (grey arrows). White dashed lines represent the animal’s border. White arrows represent the animal’s orientation. A: anterior; V: ventral. Scale bars are 20 µm. (C) Schematic of an animal highlighting the *C. elegans* coelomocytes. (D) Quantification of coelomocyte uptake of INS-1::GFP (n = 17, 13 coelomocytes). Each data point represents signal from a coelomocyte. Statistical analysis was done using the Mann Whitney U/Wilcoxon rank sum exact test. ***p ≤ 0.001.
(PDF)

S3 Fig Mutants of neuropeptide processing and release exhibit largely unaffected coelomocyte uptake of NLP-40::GFP. **(A–D)** Representative confocal images of the body of wild type and mutant animals expressing *nlp-40*::GFP in the intestine, as shown in the indicated genotype. Grey arrows indicate coelomocytes. White dashed lines represent the animal’s border. White arrows represent the animal’s orientation. A: anterior; V: ventral. Scale bars are 20 µm. (E) Schematic of an animal highlighting the *C. elegans* coelomocytes. (F) Quantification of coelomocyte uptake of NLP-40::GFP (n = 12–16 coelomocytes). Each data point represents signal from a coelomocyte. Statistical analysis was done using the Kruskal Wallis test followed by a post-hoc Steel test for pairwise comparisons with wild type. ‘ns’ indicates not significant (p > 0.05).
(PDF)

S1 Table *C. elegans* strains. (XLSX)

S2 Table Plasmids. (XLSX)

S3 Table Primer and CRISPR-related sequences. (XLSX)

S1 File Aleogho and Noma dataset. (XLSX)
