# Supplementary material for: Neuropeptide visualization using split GFP in live C. elegans
Source: PLoS One. 2026 Aug 19;21(8):e0355191. doi: 10.1371/journal.pone.0355191 (PMC13489428; doi:10.1371/journal.pone.0355191)

Supplementary Figure 1

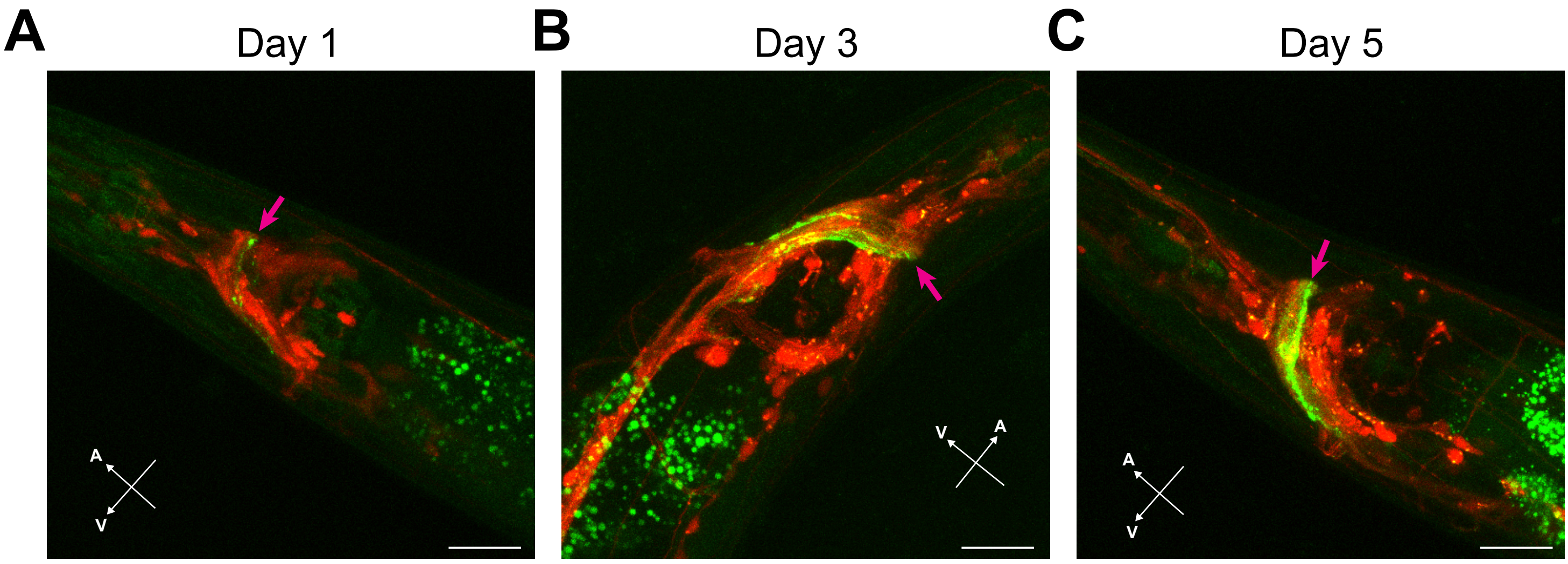

ins-1(knj35[ins-1::GFP11]); knjEx78[rgef-1p::SignalSeq-GFP1-10::CD4 + rgef-1p::TagRFP+ rol-6(su1006)]

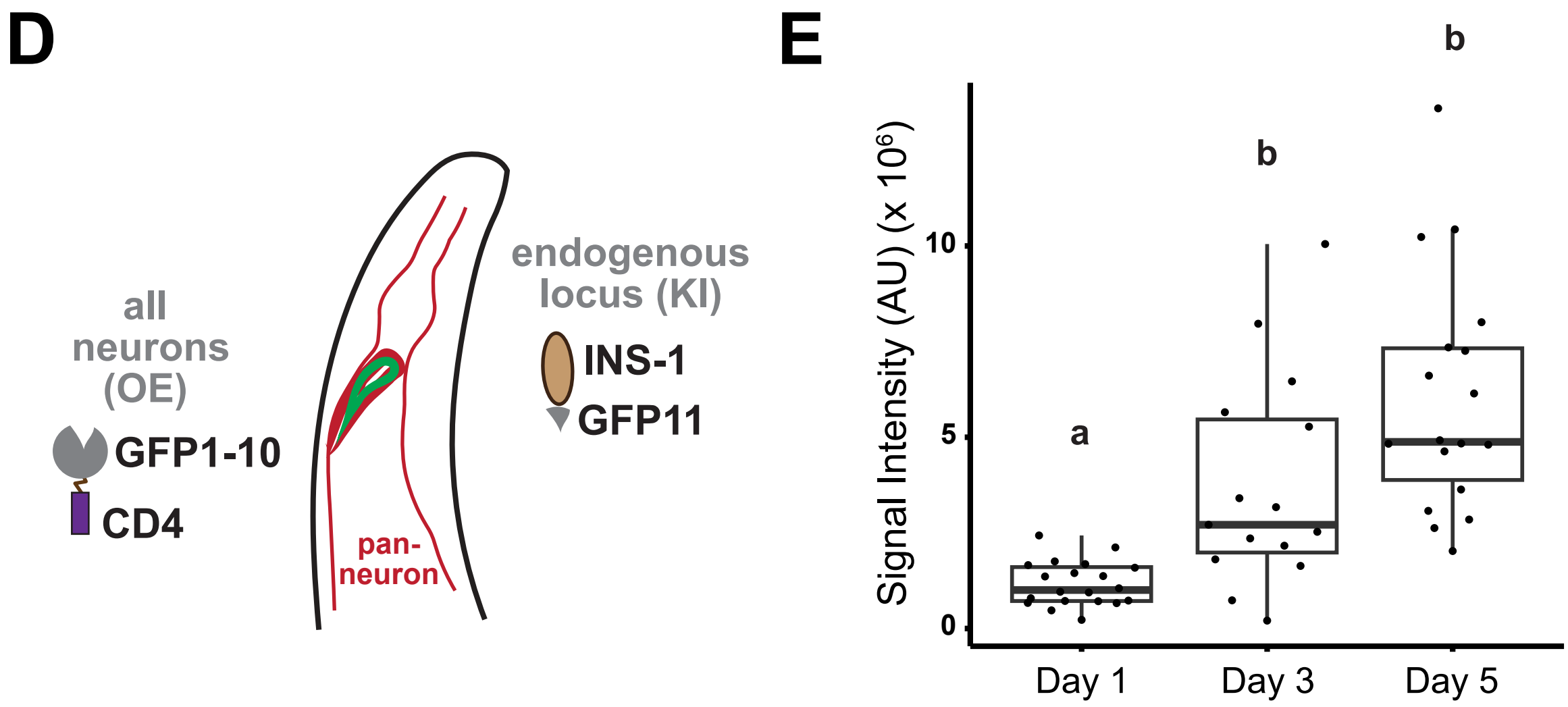

Supplement: Supplementary file 1 [file pone.0355191.s001.pdf]
