# Supplementary material for: Neuropeptide visualization using split GFP in live C. elegans
Source: PLoS One. 2026 Aug 19;21(8):e0355191. doi: 10.1371/journal.pone.0355191 (PMC13489428; doi:10.1371/journal.pone.0355191)

Supplementary Figure 2

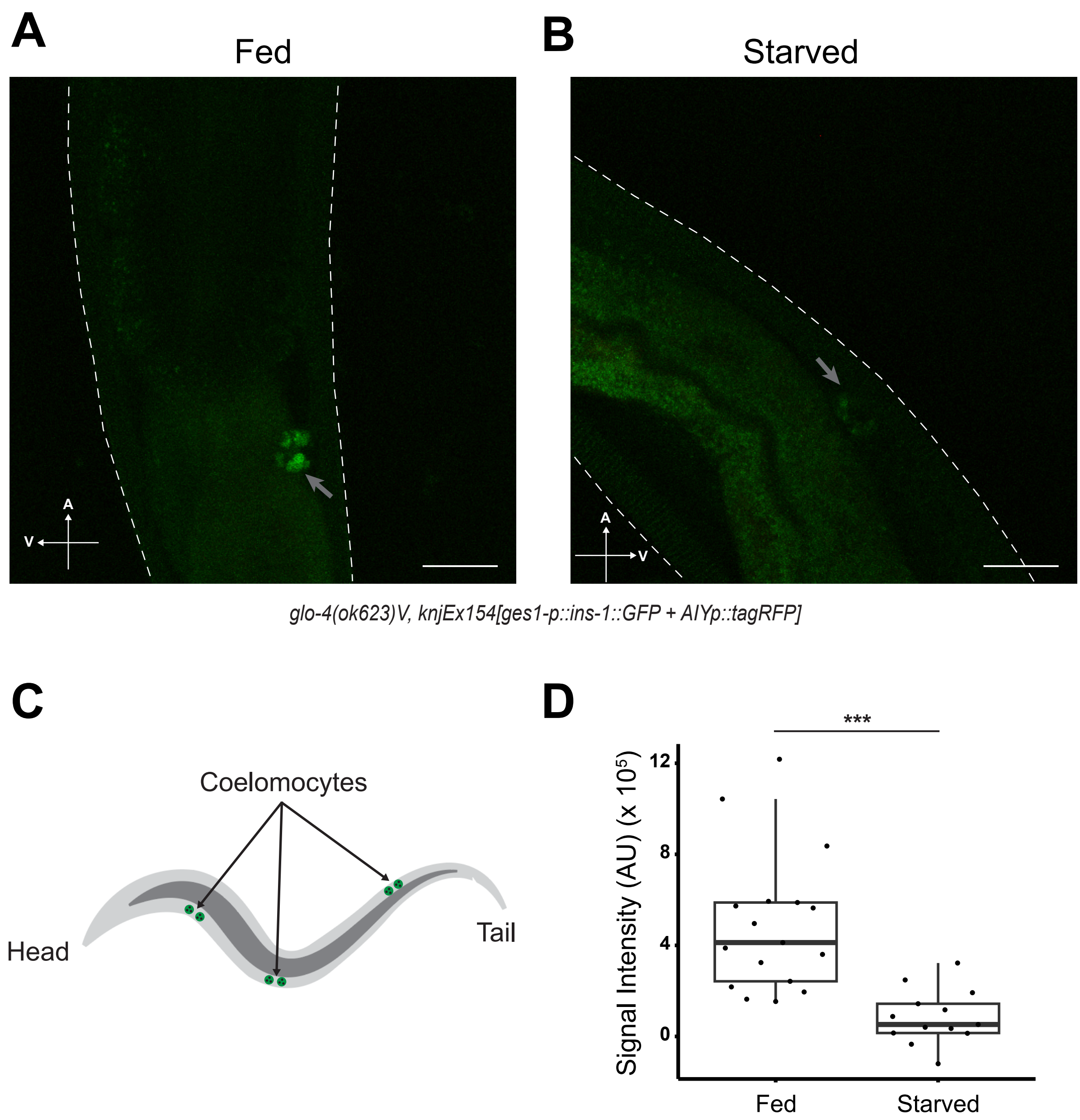

Supplement: Supplementary file 2 [file pone.0355191.s002.pdf]
