# Supplementary material for: Neuropeptide visualization using split GFP in live C. elegans
Source: PLoS One. 2026 Aug 19;21(8):e0355191. doi: 10.1371/journal.pone.0355191 (PMC13489428; doi:10.1371/journal.pone.0355191)
Supplement: Supplementary file 5 [file pone.0355191.s011.pdf]

Rebuttal letter for "Neuropeptide visualization using split GFP in live *C. elegans*" by Aleogho *et al.*, 2nd round

June 22, 2026

We appreciate the constructive feedback from the reviewers. We have revised the main texts based on the comments, but did not add any additional data in this round of revision. Our responses are shown in blue, while the original comments are in black. The cited portions of the main text are enclosed in quotation marks. All changes in the manuscript text were tracked in the tracked version.

Reviewers' comments:

Reviewer #2: I raised four concerns in the initial evaluation. The authors have sufficiently addressed points 1, 2, and 4.

Regarding point 3, I appreciate the clarification on the choice of AFD/AIY promoters. However, my concern was about if the observed unc-31-independent secretion could result from ins-1 overexpression and ectopic expression, and if altered cellular context or overexpression could potentially bypass canonical dense-core vesicle machinery. In particular, if the unc-31 mutants used retain partial activity (as discussed by the authors, 456-457), may overexpression lead to the observed phenotype. I would recommend that the authors discuss alternative possibilities and clarify if unc-31-independent secretion is context-dependent, even though it would be ideal if additional experiments, such as single copy knock-in and AIA expression, were performed.

We appreciate that the reviewer raised an important point. We did not use *ins-1* knock-in animals for mutant analyses for two reasons. First, *ins-1* is close to *egl-21* and *unc-31* on the same chromosome (IV, *ins-1*, 4.59; *egl-21*, 4.60; *unc-31*, 6.32). Second, *ins-1* knock-in produced weaker signals than *ins-1* overexpression strains. Therefore, instead of doing additional experiments using knock-in strains, we added the following discussion:

Discussion, page 20, Line 479-481
"Another limitation is that the mutant analyses relied on exogenously overexpressed INS-1 and NLP-40 using the extrachromosomal arrays, which may mask the endogenous regulation of these neuropeptides."

Reviewer #3: In this work, Aleogho et al. describe a split GFP approach aimed at labeling tissues with access to neuropeptides released from either neurons or the intestine. They show that they could detect fluorescence on the surface of cells that arises specifically from reconstitution of GFP by tagged peptides. The data supporting their conclusion are clear and well presented, and the study convincingly demonstrates that peptides released from specific cells can access the surfaces of other nearby or more distant cells and that this access occurs via a mechanism that likely involves their diffusion within the extracellular fluid following their release. The advantages and limitations of this study in detecting neurosecretion and in

quantifying neuropeptide release efficacy were demonstrated experimentally and well described.

Comments:

1) The authors focus on interactions of peptides with cells in the nerve ring and show nice images and quantification that support these interactions. They also mention interactions of peptides with neurons in the ventral nerve cord and they show some images where the anterior nerve cord can be seen (e.g. Fig 2f). It would strengthen the paper if the authors could add additional images of the nerve cord. These images would be helpful because each soma and process can be seen clearly in the same plane. Presumably, NLP-40 from the intestine should have access to these neurons. For this reason, it may be that quantification of fluorescence signal in the ventral nerve cord would be more accurate (and sensitive to changes) than in the nerve ring (error bars of nerve ring fluorescence throughout are quite large—e.g. Fig 4). Although adding images and analysis of the ventral nerve cord could strengthen the paper, adding these images/experiments may not be necessary for publication of this study.

We appreciate the reviewer's comments and suggestions. However, we decided not to include additional images. We were able to observe the nerve cord even when we focused on the nerve ring region and found similar variability and trends in both regions. Moreover, we could detect NLP-40 signals even in the anterior nerve cord and nerve ring. Therefore, we do not believe that the issue is the distance from the site of NLP-40 secretion. Instead, the major limitation is likely the use of extrachromosomal arrays. Regarding that point, we added the following sentence in Discussion (see Reviewer 2's comment as well):

Discussion, page 20, Line 479-481
"Another limitation is that the mutant analyses relied on exogenously overexpressed INS-1 and NLP-40 using the extrachromosomal arrays, which may mask the endogenous regulation of these neuropeptides."

2) Figure 4: The authors report showing no changes in fluorescence of neuropeptides expressed from the intestine in egl-21 or unc-31 mutants. One explanation for the unc-31 observation is that unc-31 is not likely to regulate neuropeptide release from the intestine (since it is expressed there at low levels and unc-31 mutants do not have defecation defects), and a possible explanation for the egl-21 observation is that defects in neuropeptide processing may not be expected to impact their release. Another potential explanation for lack of changes seen in release mutants could be that if the GFP1-10::CD4 is expressed at limiting levels, these receptors could become saturated with even when neuropeptide secretion is compromised, making changes in secretion difficult to detect. These possibilities could be added to the Discussion.

Thank you very much for clarifying the different possibilities. We agree that *unc-31* is not strongly expressed in the intestine and added the following sentence in Discussion. We did not mention the absence of defecation defect because *unc-31* is reported to have a defecation defect (Speese et al., *J. Neurosci*, 2007, PMID: 17553987).

Discussion, page 19, Line 454-456
"For instance, the lack of *unc-31*-dependency of the intestine-derived INS-1 and NLP-40 may be explained by weak or no expression of *unc-31* in the intestine [51]."

The relationship between neuropeptide processing and secretion is an important point. We added the following discussion about *egl-21*, based on the suggestion. Thank you for pointing it out.

Discussion, page 19, Line 461-462
"Regarding the *egl-21* mutant, neuropeptide processing by EGL-21 might not be required for neuropeptide secretion."

We also added the following sentence regarding the saturation of GFP1-10 as suggested.

Discussion, page 20, Line 479-481
"In addition, the limited amount of GFP1-10::CD4 may have become saturated, thereby hindering the detection of changes in neuropeptide secretion in the mutant backgrounds."
