# Supplementary material for: Neuropeptide visualization using split GFP in live C. elegans
Source: PLoS One. 2026 Aug 19;21(8):e0355191. doi: 10.1371/journal.pone.0355191 (PMC13489428; doi:10.1371/journal.pone.0355191)
Supplement: Supplementary file 6 [file pone.0355191.s009.pdf]

Reviewer Comments

Neuropeptide visualization using split GFP in live *C. elegans* The authors have submitted a thoughtfully revised and significantly strengthened manuscript. I want to commend the team for their diligent and highly constructive approach to the prior round of feedback. Developing new visualization tools to study neuropeptide dynamics is notoriously difficult, and the authors have done a wonderful job navigating the technical caveats to provide a highly useful proof-of-concept resource for the neuroscience community.

1. Overall, the authors have rigorously addressed all substantive concerns raised during the initial review. The addition of the behavioral validation (the NaCl chemotaxis assay in Figure 1J) is particularly appreciated. Demonstrating that the *ins-1::GFP11* knock-in animals retain functional associative learning—even while co-expressing the pan-neuronal *GFP1-10::CD4*—is a major milestone for this tool. Proving that a functionally tagged peptide remains biologically active is often the highest hurdle in this type of work, and this new data instills great confidence in the system's physiological relevance.
2. Furthermore, the authors handled the complex questions surrounding subcellular reconstitution versus true extracellular secretion with excellent scientific maturity. By thoughtfully tempering their claims in the Results and Discussion—and explicitly acknowledging the potential for intracellular complementation within the secretory pathway during co-expression—the manuscript is now much stronger and more transparent. The reliance on the trans-synaptic AFD-AIY model (Figure 2C) beautifully highlights the tool's true capacity to visualize extracellular peptide dispersion between distinct, anatomically separated cells.
3. I also want to highlight the elegant way the authors addressed the unexpectedly persistent signals in the secretion mutants (*unc-31*, *snt-2*). The inclusion of the new coelomocyte uptake assays (Supplementary Figures 2 and 3) was a clever and highly effective strategy. By demonstrating that bulk coelomocyte uptake remains unaffected in these genetic backgrounds, they successfully decoupled expression levels from canonical secretion dynamics, proving that the split-GFP signal reflects genuine biology rather than a mere assay artifact.
4. Finally, the candid discussion regarding the assay's current limitations—such as the sensitivity threshold for lowly expressed neuronal peptides and the high variability introduced by extrachromosomal arrays—adds tremendous practical value to the paper. Rather than over-engineering unstable lines to force a perfect result, the authors have provided a clear, honest roadmap for other labs who will undoubtedly want to adapt and optimize this tool for single-copy knock-ins in their own circuits of interest.

The authors have fully satisfied the prior critiques with rigorous new control experiments and careful, balanced textual revisions. This is a robust methods paper that will be a great asset for researchers mapping neuropeptide networks. I enthusiastically recommend this revised manuscript for publication in *PLOS One*.
