# Supplementary material for: Neuropeptide visualization using split GFP in live C. elegans
Source: PLoS One. 2026 Aug 19;21(8):e0355191. doi: 10.1371/journal.pone.0355191 (PMC13489428; doi:10.1371/journal.pone.0355191)
Supplement: Supplementary file 7 [file pone.0355191.s010.docx]

**Rebuttal letter for “Neuropeptide visualization using split GFP in live *C. elegans*” by Aleogho *et al*.**

February 8, 2026

We appreciate the constructive feedback from the editor and reviewers and have substantially revised the manuscript based on the comments. Our responses are shown in blue, while the original comments are in black. The cited portions of the main text are enclosed in quotation marks and underlined. All changes in the manuscript text were tracked. Some figure panels were replaced and new figures were added. Please note that in our responses below, any of such references refer to the revised manuscript.

We are glad the reviewers found our study interesting and offered constructive comments. As the reviewers suggested, we added new experiments [Reviewer #1 (points 1 and 2) and Reviewer #3 (points 3 and 4)] and clarified some points in the Introduction, Results, and Discussion.

The following new data were added:

- Supplementary Figure 1
- Supplementary Figure 2
- Supplementary Figure 3

The following figures were replaced:

- Figure 1J
- Figure 3E
- Figure 3G

**Editor’s Comments:**

Journal Requirements:

When submitting your revision, we need you to address these additional requirements.

1. Please ensure that your manuscript meets PLOS ONE's style requirements, including those for file naming. The PLOS ONE style templates can be found at

<https://journals.plos.org/plosone/s/file?id=wjVg/PLOSOne_formatting_sample_main_body.pdf> and

[https://journals.plos.org/plosone/s/file?id=ba62/PLOSOne_formatting_sample_title_authors_affiliations.pdf](https://track.editorialmanager.com/CL0/https:%2F%2Fjournals.plos.org%2Fplosone%2Fs%2Ffile%3Fid=ba62%2FPLOSOne_formatting_sample_title_authors_affiliations.pdf/1/010f01997d68fd4d-fdc0c316-6cf8-42bf-9fa1-bafa0f471815-000000/myQgZw3UufKnQUw42NiPQjVMowummNWJ2WUxJfq73dU=230).

We have carefully formatted the manuscript according to PLOS ONE’s style requirements. We effected the following changes:

- Converted the manuscript to double-space paragraph format
- Moved figure captions to appear after the paragraphs in which they’re first mentioned
- Named files appropriately

2. Thank you for stating in your Funding Statement:

“K.N. received the awards.

This work was supported by MEGMILK SNOW BRAND company and JST FOREST Program, Grant Number JPMJFR 214V.

The funders did not play any role in the study design, data collection and analysis, decision to publish, or preparation of the manuscript.”

Please provide an amended statement that declares *all* the funding or sources of support (whether external or internal to your organization) received during this study, as detailed online in our guide for authors at <http://journals.plos.org/plosone/s/submit-now>.  Please also include the statement “There was no additional external funding received for this study.” in your updated Funding Statement.

Please include your amended Funding Statement within your cover letter. We will change the online submission form on your behalf.

Thank you. We have provided an amended Funding Statement on the online submission system, cover letter, and manuscript.

3. Thank you for stating the following in your manuscript:

“This work was supported by MEGMILK SNOW BRAND company and JST FOREST Program, Grant Number JPMJFR 214V.**”**

We note that you have provided additional information within the Acknowledgements Section that is not currently declared in your Funding Statement. Please note that funding information should not appear in the Acknowledgments section or other areas of your manuscript. We will only publish funding information present in the Funding Statement section of the online submission form.

Please remove any funding-related text from the manuscript and let us know how you would like to update your Funding Statement. Currently, your Funding Statement reads as follows:

“K.N. received the awards

This work was supported by MEGMILK SNOW BRAND company and JST FOREST Program, Grant Number JPMJFR 214V.

The funders did not play any role in the study design, data collection and analysis, decision to publish, or preparation of the manuscript.”

Please include your amended statements within your cover letter; we will change the online submission form on your behalf.

We apologize for the oversight. We have removed the funding-related text from the Acknowledgements sections and added it to the amended Funding section on the manuscript, online Funding Statement, and cover letter.

4. We notice that your supplementary tables are included in the manuscript file. Please remove them and upload them with the file type 'Supporting Information'. Please ensure that each Supporting Information file has a legend listed in the manuscript after the references list.

We have removed the tables from the manuscript and uploaded them with the ‘Supporting Information’ file type.

5. If the reviewer comments include a recommendation to cite specific previously published works, please review and evaluate these publications to determine whether they are relevant and should be cited. There is no requirement to cite these works unless the editor has indicated otherwise. 

We note this and thank the editor. After evaluation, we included the reference Kurashina *et al*, 2025 suggested by Reviewer #2 (point 2). (Ref. 36. Page 3, line 71).

**Reviewers' comments:**

Reviewer's Responses to Questions

**Comments to the Author**

1. Is the manuscript technically sound, and do the data support the conclusions?

The manuscript must describe a technically sound piece of scientific research with data that supports the conclusions. Experiments must have been conducted rigorously, with appropriate controls, replication, and sample sizes. The conclusions must be drawn appropriately based on the data presented.

Reviewer #1: Partly

Reviewer #2: Partly

2. Has the statistical analysis been performed appropriately and rigorously?

Reviewer #1: Yes

Reviewer #2: Yes

3. Have the authors made all data underlying the findings in their manuscript fully available?

The [PLOS Data policy](http://track.editorialmanager.com/CL0/http:%2F%2Fwww.plosone.org%2Fstatic%2Fpolicies.action%23sharing/1/010f01997d68fd4d-fdc0c316-6cf8-42bf-9fa1-bafa0f471815-000000/XVWHG085ogOtm0L7caOu6-SxBuWmCTn3s7SOhR3Uua0=230) requires authors to make all data underlying the findings described in their manuscript fully available without restriction, with rare exception (please refer to the Data Availability Statement in the manuscript PDF file). The data should be provided as part of the manuscript or its supporting information, or deposited to a public repository. For example, in addition to summary statistics, the data points behind means, medians and variance measures should be available. If there are restrictions on publicly sharing data—e.g. participant privacy or use of data from a third party—those must be specified.

Reviewer #1: Yes

Reviewer #2: Yes

4. Is the manuscript presented in an intelligible fashion and written in standard English?

PLOS ONE does not copyedit accepted manuscripts, so the language in submitted articles must be clear, correct, and unambiguous. Any typographical or grammatical errors should be corrected at revision, so please note any specific errors here.

Reviewer #1: Yes

Reviewer #2: Yes

5. Review Comments to the Author

Please use the space provided to explain your answers to the questions above. You may also include additional comments for the author, including concerns about dual publication, research ethics, or publication ethics. (Please upload your review as an attachment if it exceeds 20,000 characters)

**Reviewer #1:**

Aleogho and Noma report a novel method for observing neuropeptides and their secretion. They used a split GFP, which allows the observation of neuropeptide binding to a non-endogenous receptor (CD4). They show that tagging two neuropeptides (INS-1 and NLP-40) with the GFP11 fragment doesn’t interfere with their function.
This technique is interesting and promising to better understand how neuropeptides are secreted and interact with receptors.

We are glad that the reviewer finds this work interesting and promising.

I do have some questions about the study:

Major:
1. You state in the introduction that the interaction between GFP11 and GFP1-10 is irreversible (p2 line 68), I would agree with that statement. However, this brings some concerns about how it could affect the animals as it means the neuropeptide would bind “forever” to its receptors (here CD4, but in further study, endogenous receptor). It would be interesting to confirm this by doing some dynamic studies or seeing the evolution of GFP with age.

We thank the reviewer for this comment and agree that the stable interaction between GFP11 and GFP1–10 may perturb the original signaling from a tagged neuropeptide to its receptor. To reduce the distortion due to irreversible binding, we may be able to use a split fluorescent tag that allows reversible binding (PMID: 31249300). It was not our first choice because we would sacrifice the intensity. Further optimization will be necessary in the future. We added discussion regarding these matters as shown below:

“Another limitation of the split GFP is the irreversible binding of GFP11 and GFP1-10, which might perturb endogenous signaling dynamics. Future optimization may address this limitation through the use of alternative split fluorescent systems with reversible complementation.” (Discussion, Page 21, lines 487-490).

Furthermore, to directly address the reviewer’s concern regarding signal dynamics, we analyzed the change of GFP fluorescence with age (Supplementary Figure 1). We observed a progressive increase in GFP signal from Day 1 to Day 5, consistent with cumulative detection of interaction events over time rather than sustained receptor engagement. This observation is expected for irreversible GFP complementation. The main text was revised accordingly:

“Since the binding between INS-1::GFP11 and GFP1-10::CD4 is thought to be irreversible, the observed signals likely reflect the accumulation of past secretion events. To assess whether split GFP signal accumulates over time, we quantified fluorescence intensity of *ins-1*::GFP11 KI and pan-neuronal GFP1-10::CD4 at different adult ages (S1A–D Fig). GFP signal increased progressively from Day 1 to Day 5 (S1E Fig), consistent with integration of reconstituted GFP signals.” (Results, Page 7, line 167 – Page 8, line 172)

2. Were the behavior assays (defecation, salt conditioning) executed with the expression of GFP1-10::CD40 or just the tagging (GFP11) of the neuropeptides? In the legend and the text, it only talks about neuropeptide tagging. It is essential to test it with GFP1-10::CD40 expression, also to show that having the neuropeptides being taken away from their endogenous receptors doesn’t interfere with the function of those neuropeptides, which could explain some of the rescue results (aka no rescue).

We thank the reviewer for raising this concern. The reviewer is correct; in the original manuscript, we executed behavioral assays with strains expressing just the tagging (GFP11) of the neuropeptides to address if tagging itself perturb the functionality. Based on the reviewer’s suggestion, we conducted an additional experiment to see whether co-expression of GFP1–10::CD4 and *ins-1*::GFP11 affects behavioral outputs by performing NaCl chemotaxis assays. As shown in Figure 1J, animals co-expressing *ins-1*::GFP11 and GFP1–10::CD4 displayed chemotaxis indices that were not significantly different from wild-type controls in the Naïve or Mock-conditioned groups and showed the expected reduction in chemotaxis following starvation conditioning with NaCl. These results indicate that co-expression of *ins-1::*GFP11 and GFP1–10::CD4 does not impair NaCl chemotaxis or learning and therefore does not measurably perturb this behavioral output. We have clarified these points in the Results section to explicitly state which behavioral assay was performed in the presence of GFP1–10::CD4:

“The *ins-1*::GFP11 KI animals behaved similarly to the wild type across all three groups (Fig 1J), indicating normal function of GFP11-tagged INS-1. Moreover, co-expression of GFP1–10::CD4 with ins-1::GFP did not significantly alter NaCl chemotaxis in the Naïve or Mock-conditioned groups and only slightly prevented learning-induced changes in chemotaxis index following NaCl-conditioning (Fig 1J).” (Results, Page 8, lines 180-184)

For the defecation motor program assay, we did not repeat the experiment using GFP1–10::CD4–expressing strains. This decision was based on the consideration that GFP11 tagging likely interfered with the function and/or proper cleavage of P3 peptide (Figure 3G. We discussed further in Major point 6). We independently repeated the original defecation assay with an increased sample size to clarify the initial result. This repeat experiment reproduced the original trend (Figure 3G), supporting the robustness of the conclusion. Given these considerations, we chose not to pursue additional defecation assays with GFP1–10::CD4 strains.

3. In the first part of the study, GFP1-10::CD40 and INS-1::GFP11 are expressed in the same cells (Fig1). It would be important to indicate that the signal observed could be due to their interaction in the same cells, including intracellular binding.

We thank the reviewer for raising that point and have indicated the limitation in the Results section as shown below:

“Because the same neurons might express both GFP1–10::CD4 and *ins-1*::GFP11 in both configurations in Fig 1C–E, we could not exclude the possibility that the reconstituted GFP signals might originate from intracellular compartments such as ER, Golgi, and endosomes in addition to potential extracellular interactions.” (Page 7, lines 163-166)

4. Fig 2: The image H (starved) looks like the GFP signal is less diffused than in G(fed). Is this representative? Would it be supported by the known role of INS-1 during starvation? Could the difference be tested (number, size, and/or intensity of the puncta observed)?

We thank the reviewer for this comment. Figure 2G (fed) and H (starved) show representative examples of intestine-derived INS-1::GFP11 signal. However, across animals, we did not observe a consistent starvation-dependent change in the overall distribution or appearance of the reconstituted GFP signal. Although, there was a trend of increase, quantification of signal intensity (Fig. 2J) showed no significant difference between fed and starved conditions.

We interpret the apparent difference in signal “diffuseness” between the individual images in Figure 2G and H as falling within normal biological and imaging variability, rather than reflecting a robust starvation-dependent change in INS-1 localization. Because no consistent qualitative difference was observed across animals, we did not further quantify puncta number, size, or intensity, but we mentioned this observation in the Results as follows:

“Compared to well-fed animals, animals starved for 3 hours exhibited a trend of increase in reconstituted GFP signal along the nerve ring, while we did not see any consistent changes in the localization patterns (Fig 2G–J). ” (Page 11, lines 246-248).

5. Fig 3 G: Can you explain/ give hypotheses of why the rescue is better in the glo-4 background?

We are not sure, but there could be genetic interaction between the *glo-4* and *nlp-40* mutant backgrounds given that both are expressed in the intestine. For simplification and clarity, we removed the *glo-4* data and tested more samples of the wild type to validate the previous data. Please see the new Figure 3G. We have also edited the text accordingly.

6. Fig 3G: Can you explain and give more details and hypotheses for the lack of rescue in the defecation assay? Is it due to the expression go NPL-40 in the gut, and maybe neuron rescue is also necessary for this behavior? Or could the GFP interfere with P3 binding to its AEX-2 receptor? Could the P3 and P4 tagging be done as a knock-in instead of overexpression to avoid mosaic expression?

We thank the reviewer for these thoughtful suggestions. We note that in the previous paper by Wang *et al* (PMID: 23583549), the expulsion defect of *nlp-40* mutants was fully rescued by overexpression of *nlp-40*. However, in Figure 3G of our study, overexpression of intestinal *nlp-40(P3)*::GFP11 or *nlp-40(P4*)::GFP11 in the *nlp-40* mutant background does not robustly restore expulsion frequency to wild-type levels.

In our *nlp-40(P3*)::GFP11 and *nlp-40(P4*)::GFP11 constructs, the full *nlp-40* precursor is present, and all NLP-40–derived peptides are produced; only a single peptide (P3 and P4, respectively) carries the GFP11 tag. Therefore, the lack of robust rescue is not due to the absence of other NLP-40 peptides. Instead, several non-mutually exclusive factors may contribute to the incomplete rescue. First, effective restoration of expulsion may require endogenous-level expression and tightly regulated secretion dynamics, which are difficult to reproduce with extrachromosomal overexpression and may be further affected by mosaic expression. Second, although GFP11 is a minimal tag, tagging the precursor at a specific peptide position could cause steric effects and affect peptide processing, trafficking, or effective availability of the tagged peptide, thereby reducing overall functional output. This could explain the absence of rescue using the *nlp-40(P3*)::GFP11 construct. Third, expulsion behavior is known to integrate multiple signals and physiological states; consequently, restoring intestinal NLP-40 signaling alone, even with an otherwise intact precursor, may be insufficient to fully reconstitute normal expulsion frequency. We emphasized the second point to the main text:

“Although, there was no rescue of the defecation phenotype in *nlp-40(tm4085)* mutants carrying the *nlp-40*(P3)::GFP11 transgene, those carrying the *nlp-40*(P4)::GFP11 transgene displayed an expulsion phenotype intermediate to those of the wild type and *nlp-40(tm4085)* mutants (Fig 3G). We note that the *nlp-40*(P4)::GFP11 strain should express untagged *nlp-40*(P3) (Fig 3A). These results indicate that GFP11 likely interfered with the function and/or proper cleavage of the rescuing P3 peptide and support the results from the previous study showing the necessity and sufficiency of the P3 peptide in *C. elegans* defecation [45].” (Results, Page 13, lines 312-318)

We agree that a CRISPR knock-in strategy would minimize mosaicism and preserve endogenous expression levels and would therefore be an elegant approach to address these issues. However, we did not prioritize this additional experiment, given that tagging *nlp-40*(P3) with GFP11 did not rescue the defecation phenotype of *nlp-40* mutants.

7. Fig 4: Some of the results are quite variable, which may be due to the mosaicism of extrachromosomal lines. It often happens that the GFP expression of extrachromosomal arrays changes after crossing. It would be better to do those experiments in integrated lines, which would have a more stable expression. The KI of GFP11 would be a great way to control the expression, too, with integrated CD40.

We agree with the reviewer that mosaicism associated with extrachromosomal arrays can contribute to variability, and that integrated lines or knock-in strategies would, in principle, provide more stable expression.

We therefore generated an integrated GFP1–10::CD4 transgene and tested it in combination with the *ins-1*::GFP11 knock-in background. However, under these conditions, we were unable to detect any GFP signal, despite robust expression of the reporters possibly due to the background mutations generated during integration of GFP1-10::CD4.

Importantly, despite the use of extrachromosomal expression, the qualitative conclusions of our study are supported by consistent trends observed across multiple constructs, and by complementary assays that do not rely on split GFP reconstitution e.g., whole GFP–based coelomocyte uptake assays (Supplementary Figures 2 and 3). We therefore think this strategy is a practical compromise between signal detectability and expression stability. We agree that knock-in–based approaches aimed at optimizing split GFP sensitivity at endogenous expression levels represent an important future direction and mentioned it in Discussion as shown:

“A limitation of this study is the reliance on extrachromosomal arrays to express GFP1–10 constructs, which can introduce mosaicism and contribute to variability. Moreover, the method using GFP1–10::CD4 primarily reports peptide release and local dispersion near target membranes, rather than engagement to cognate receptors. Future development of knock-in–based split-GFP strategies using GFP1-10-tagged cognate receptors rather than CD4 will be important for extending this approach under fully physiological conditions to map neuropeptide signal transduction *in vivo*.” (Page 20, lines 479-485).

8. Those results (Fig4) showing an increase in fluorescence are really interesting and are worth exploring further. Does the signal look more diffused? Less localized at the synapse, maybe? I guess with CD4 being a non-endogenous receptor means it is not localized at the synapses, and could explain the diffusion of the signal. This is where it would be good to expand the discussion on this part, especially given the results from Wang et al (2013). They demonstrated that in the snt-2 mutant, NPL-40 is less secreted due to a lower intensity of YFP from the NLP-40 transgenic reporter in the coelomocytes.

We thank the reviewer for the comment and helpful suggestion. The signal in Figure 4 does not look more diffused. In Figure 4G–L, the reconstituted split GFP signal reflects proximity between INS-1::GFP11 and membrane-tethered GFP1–10::CD4, not a synapse-restricted postsynaptic receptor readout. Because CD4 is a non-endogenous membrane anchor and is not confined to synaptic specializations, the signal can appear comparatively diffuse and should be interpreted as peptide arrival/accumulation near the target cell surface rather than precise synaptic localization.

With respect to Wang *et al.* (2013), their coelomocyte assay reports **bulk secretion of NLP-40** and showed reduced coelomocyte fluorescence from an ***nlp-40p::nlp-40::YFP*** reporter in *snt-2* mutants. To relate our study to this framework, we performed an independent coelomocyte uptake assay using **intestine-expressed *ges-1p::nlp-40::GFP*** and found no significant change in coelomocyte signal across *unc-31*, *snt-2*, and *egl-21* mutants (Supplementary Figure 3), indicating that—under our assay conditions—bulk pseudocoelomic accumulation from the intestinal reporter is not increased in *snt-2*. This could be due to the difference in promoter.

Thus, the elevated fluorescence in Figure 4 (INS-1 split GFP) is not evidence for increased global secretion but instead is most consistent with altered local peptide availability/retention near target membranes when release/clearance dynamics are perturbed. We have expanded the Discussion to clarify these assay-dependent readouts and how they complement the bulk-secretion measurements in Wang et al. (2013):

“Our findings for NLP-40 should also be considered in relation to prior bulk secretion assays. Wang et al. observed reduced coelomocyte fluorescence of NLP-40 in *snt-2* mutants [45]. However, we found no significant change in coelomocyte signal across *snt-2* and other mutants (**S3 Fig**). While this difference may reflect assay conditions or promoter-specific expression, elevated fluorescence in the split GFP configuration (Fig 4G–L) may reflect increased local peptide availability near the nerve ring, rather than increased global secretion.” (Page 18, line 437 – Page 19, line 443).

Minor:
1. Can you explain why the glo-4 background could not be isolated in some strains?

It could have been due to human error during crossing. We repeated the crossing and were able to isolate *glo-4* background for the *nlp-40*(P4)::GFP11 which is now the new Figure 3E.

2. Line 225, I assumed you used the non-transgenic siblings as a control, or did you isolate a line from non-transgenic siblings before evaluation? Could you clarify?

We used non-transgenic siblings as a control without isolating non-transgenic lines. We have stated this as follows:

“As negative controls, we also evaluated the non-transgenic siblings and observed no rescue (Fig 3G).” (Results, Page 13, line 318 – Page 14, line 320).

3. In Fig 2F, a picture of the ventral nerve cord expression is missing. You state in the text that you can observe GFP in the nerve cord, but you do not support that claim with an image in the figure. Would it be possible to add one?

We apologize that it was unclear. The ventral nerve cord is indicated by the yellow arrows.

4. Recently, the use of rol-6 as a co-injection marker has been shown to affect synaptic phenotypes in male neurons (Jin et al (2019, 10.1371/journal.pone.0224533). Even though this study focuses on males, it would be good to talk about this caveat in the results, so people can choose a different co-injection marker if they wish.

We thank the reviewer for the information and have included this caveat and cited the paper. Since we could not find a suitable place to discuss this point in the Results, we included it in the Materials and Methods:

“Although *rol-6(su1006)* has been reported to influence synaptic phenotypes in male-specific neurons, all genotypes within each experiment used the same co-injection marker, reducing the likelihood of marker-specific effects; future work could avoid this caveat by using alternative markers or single-copy knock-in approaches.” (Materials and Methods, Page 22, lines 521-524)

**Reviewer #2:**

In this study, Aleogho et al. applied the split GFP system for in vivo visualization of neuropeptide release in C. elegans, focusing on INS-1 and NLP-40. The advantage of this approach is that tagging neuropeptide with GFP11 minimizes the functional disruption of neuropeptide compared to full-length fluorescent tags. It allows detection of GFP11-tagged neuropeptide at sites where GFP1-10 is expressed. The paper is straightforward and easy to follow. The observation that INS-1 and NLP-40 secretion from AFD and intestine was not inhibited in unc-31, snt-2, or egl-21 is unexpected and interesting, and possibly worth further exploring.

We are glad that the reviewer finds our paper interesting, straightforward, and easy to follow.

I have a few concerns:
1. Methods: The manuscript does not provide sufficient information on construct design and experimental procedures. For instance, the DNA templates used for GFP11 knock-ins, the signal sequences used for GFP1-10 targeting, and primer sequences for cloning are not described. In the chemotaxis assay, the author indicated n = 7 but it remains unclear if these assays were 7 technical replicates or from different biological replication. It remains unclear by checking the source data. It is also unclear if the displayed images were the maximum projections of z-stacks or taken from a single focal plane.

We apologize for the insufficient information and have provided additional information to the Supplementary information regarding the DNA template for GFP11 knock-ins, the signal sequences used for GFP1-10 targeting, and primer sequences for cloning. In the chemotaxis assay, ‘n’ indicates an independent assay plate. The behavioral experiments were conducted with 1~3 assay plates per day on at least three different days to check the reproducibility of the trend, and we pooled these data. We clarified this in the Materials and Methods section as follows and included what “n” means in all the figure legends:

“Experiments were conducted on at least three different days to check the reproducibility of the trend, and each data point represents an independent assay plate.” (Materials and Methods, Salt chemotaxis assay, Page 23, lines 549-551)

The images we provided are all maximum-intensity projections, which is clarified as follows:

“Displayed images are maximum-intensity projections of Z-stacks.” (Materials and Methods, Fluorescence microscopy, Page 22, lines 532-533)

2. References: A recent publication by Kurashina, using split GFP and wrmScarlet systems to label endogenous presynaptic protein, was not cited.

We apologize for the oversight and have cited the paper (Ref 36, Page 3, line 71).

3. Neurons: Could authors explain why they choose AFD neurons, in which ins-1 does not seem to be endogenously expressed? Would this, and intestinal overexpression, underlie the unexpected secretion of two neuropeptides in different mutants? Would it be better if the ins-1::GFP11 knock-in strain or different neurons such as AIA were used?

We thank the reviewer for this question. In this experiment, we wanted to demonstrate that GFP11-tagged neuropeptide and GFP1–10::CD4 are reconstituted outside of the cell. When we use the endogenous *ins-1*::GFP11 knock-in, we cannot exclude the possibility that *ins-1*::GFP is reconstituted with GFP1–10-expressing cells. Although we could use alternative neurons such as AIA, we used AFD and AIY pair because their promoters (*gcy-8p* and *ttx-3p*, respectively) are very exclusive in our hands and suitable to exclude the possibility that *ins-1*::GFP11 and GFP1–10 are expressed in the same neurons. To further address the endogenous regulation of the neuropeptide, we agree that we should use other constructs like *ins-1*::GFP11 knock-in and AIA neurons.

We have clarified this rationale in the Results to avoid confusion regarding endogenous *ins-1* expression and to emphasize that this experiment is designed to assess the reconstitution outside of the cell rather than native expression patterns.

“Although AFD does not endogenously express *ins-1* [44], AFD and AIY neuron promoters are highly specific and non-overlapping, allowing us to exclude the possibility of intracellular GFP assembly within the same cell. ” (Results, Page 9, lines 198-201)

4. It is worth discussing how the method could be used to map neuropeptide signal transduction in physiologically relevant contexts.

Thank you for raising an important direction. We agree with the reviewer that an important future application of the split GFP approach will be to map neuropeptide signal transduction in physiologically relevant contexts. In its current form using GFP1–10::CD4, the method focuses on the release of a peptide and “possible” dispersion of the peptide.

In physiologically relevant settings, this approach could be combined with endogenous or near-endogenous expression strategies (e.g., knock-in tagging of neuropeptides with GFP11), endogenous or cell-specific expression of GFP1–10-tagged cognate receptor, and behavioral or physiological readouts to link peptide delivery to function. For example, restricting GFP1–10 to defined neuronal or non-neuronal targets would allow systematic mapping of potential sites of peptide action, while parallel genetic or environmental manipulations (such as starvation or circuit perturbations) could reveal state-dependent changes in peptide signaling.

We have added a discussion to highlight these potential applications, while emphasizing that further technical optimization will be required to adapt the method for fully endogenous and physiological contexts:

“A limitation of this study is the reliance on extrachromosomal arrays to express GFP1–10 constructs, which can introduce mosaicism and contribute to variability. Moreover, the method using GFP1–10::CD4 primarily reports peptide release and local dispersion near target membranes, rather than engagement to cognate receptors. Future development of knock-in–based split-GFP strategies using GFP1-10-tagged cognate receptors rather than CD4 will be important for extending this approach under fully physiological conditions to map neuropeptide signal transduction *in vivo*.” (Discussion, Page 20, lines 479-485)

“While additional technical optimization will be required, these extensions highlight the potential of split GFP–based strategies for dissecting neuropeptide signaling in physiologically relevant settings. Coupling this approach with behavioral, physiological, or state-dependent manipulations (e.g., starvation or circuit perturbations) would further allow peptide delivery to be linked to functional outcomes.” (Discussion, Page 21, lines 490-494)

Reviewer #3:

Aleogho et al. developed a methodology to visualize the released neuropeptides using a split GFP system and quantified their release under different conditions, including fed vs. starved states and in various mutant backgrounds.

Main criticism:
Given the current limitation of methodologies to monitor neuropeptide release, this work holds substantial potential. Nonetheless, there are some unexpected findings, and it is not clear if the methodology is sufficiently benchmarked using the reported examples:
NLP-40 and INS-1 are not affected by mutations that should influence neuropeptide release (unc-31, snt-2). This may be a specific feature of these two neuropeptides, that are released from a large tissue, i.e. the intestine, in addition to other cells. The authors should provide an example of a purely neuronally released neuropeptide, possibly from the FMRFamide class, that is known to be affected by unc-31 for its release (several examples can be found in the literature, e.g. NLP-21 or FLP-1). To thus see if this can be recapitulated by their assay, i.e. no release in such a mutant and thus no signal.

We thank the reviewer for this thoughtful and constructive suggestion. We agree that benchmarking the split GFP assay using a purely neuronally released neuropeptide whose secretion is well established to depend on UNC-31 would be a valuable way to further validate the approach.

Based on the reviewer’s suggestion, we generated cholinergic-neuron specific NLP-21::GFP strain. However, we did not observe robust signals probably due to low expression levels and thus could not test if the signals depend on *unc-31*.

In the present study, our aim was to establish proof-of-principle that split GFP reconstitution can be used to visualize neuropeptide delivery to defined target membranes *in vivo*. We therefore focused on NLP-40 and INS-1, which are well-characterized peptides with known roles in inter-tissue and neuron–target communication, and which allowed us to test the assay across intestinal and neuronal sources.

Importantly, the observation that NLP-40 and INS-1 signals are not uniformly reduced in *unc-31* or *snt-2* mutants does not necessarily indicate insensitivity of the assay. Instead, it may reflect biological features of these peptides, including their production in the intestine and the possibility that their delivery to target membranes is less strictly dependent on canonical neuronal dense-core vesicle release machinery.

We have revised the Discussion to clarify the current benchmarking limits of the assay and to explicitly outline the use of neuronally restricted, UNC-31–dependent peptides as an important future direction:

“The observation that INS-1 and NLP-40 signals are not uniformly reduced in *unc-31* or *snt-2* mutants might reflect biological features of these peptides, including their production in large non-neuronal tissues such as the intestine is less strictly dependent on canonical neuronal dense-core vesicle release machinery. Testing purely neuronally released, UNC-31–dependent neuropeptides such as FMRFamide-related peptides (e.g., NLP-21 or FLP-1) would provide an important additional benchmark.” (Discussion, Page 19, line 460 – Page 20, line 466).

Generally, the authors should also probe the limits of their approach by choosing a neuropeptide that is expressed in very few cells and at low expression level, to see if this can nonetheless be picked up by this approach. Using neuropeptides with high expression level in large cells, that will inevitably produce high amounts of secreted peptide, are probably more likely to produce detectable signal fom GFP reconstitution.

We agree with the reviewer that probing the sensitivity limits of the split GFP approach using neuropeptides expressed in very few cells and at low levels would be informative. Indeed, we could not detect stable signals for NLP-21::GFP11 from cholinergic neurons as mentioned above. In the present study, we focused on neuropeptides with relatively robust expression as a proof-of-principle to establish that split GFP reconstitution can reliably report peptide delivery to defined target membranes.

We therefore view the current data as establishing a benchmark for what can be detected with the present implementation of the system. Extending the approach to rarer or weakly expressed neuropeptides, potentially in combination with further technical optimization, represents an important future direction. We highlighted this caveat in Discussion as follows:

“Future development of knock-in–based split-GFP strategies using GFP1-10-tagged cognate receptors rather than CD4 will be important for extending this approach under fully physiological conditions to map neuropeptide signal transduction in vivo. In this case, the expression level of the targeted neuropeptides should be considered because split GFP system will sacrifice the signal intensity compared to the full-length GFP.” (Discussion, Page 20, line 482 – Page 21, line 487)

In addition, several key statements in the manuscripts are not sufficiently supported by robust evidence. I outline major and minor concerns below.

Major issues:
1. In Figure 1, CD4-GFP-1-10 and GFP11 are both expressed pan-neuronally. In Figure 2G-J, CD4-GFP-1-10 and GFP11 are supposed to be both expressed in many neurons, given the widespread expression of ins-1. Is GFP reassembled really on the extracellular surface of the cells, rather than inside ER?

We thank the reviewer for raising this important point. Indeed, because both GFP1–10::CD4 and INS-1–GFP11 are expressed broadly in neurons, GFP complementation could in principle occur intracellularly, including within the secretory pathway like rough ER and Golgi.

We think at least the signals on the nerve ring are likely outside the cells because the nerve ring is devoid of rough ER markers (PMID: 12006669). Also, we could detect extracellular signals between AFD and AIY, which excludes the possibility that *ins-1*::GFP11 and GFP1–10 are expressed within the cell (Figure 2A-C). Thus, we believe that at least a part of the signals in the nerve ring originates from the reconstituted split GFP outside the cell.

Importantly, we do not claim that Figures 1C–E and 2G–J exclusively report extracellular ligand–receptor interactions. Rather, in these configurations, split GFP signal may include contributions from intracellular compartments. For this reason, these experiments are interpreted as demonstrating feasibility and sensitivity of the assay, rather than precise subcellular localization of interaction.

We have now clarified this limitation in the Results and Discussion and avoid interpreting the signal as definitive evidence of extracellular binding:

“Because the same neurons might express both GFP1–10::CD4 and ins-1::GFP11 in both configurations in Fig 1C–E, we could not exclude the possibility that the reconstituted GFP signals might originate from intracellular compartments such as ER, Golgi, and endosomes in addition to potential extracellular interactions.” (Results, Page 7, lines 163-166)

“Second, this study could not rule out the presence of reconstituted intracellular GFP signals either during synthesis or after endocytosis, and such signals may not respond to regulation as expected. Especially when both GFP1–10::CD4 and GFP11-tagged neuropeptides are expressed in the same cells, GFP complementation could occur intracellularly within the secretory pathway, rather than exclusively at the cell surface. Accordingly, experiments in which GFP fragments are broadly co-expressed (e.g., Fig 1C–E) are interpreted as demonstration of assay feasibility and sensitivity. Nonetheless, our observations support reconstitution after the secretory pathways because GFP signals were predominantly observed along neuronal processes, which are devoid of rough ER markers [59], rather than in perinuclear regions. Especially in the experiments involving anatomically distinct source and target cells (e.g., AFD–AIY), extracellular reconstitution is strongly supported.” (Discussion, Page 20, lines 467-477)

2. Many statements are based on results without significant differences. e.g. Figures 2J, 3I, 4L. In such cases, the corresponding interpretations should be reconsidered or removed from the main conclusions.

We apologize for the overstatement. We have rephrased the statements about the figures accordingly.

3. Regarding Figure 2J, does starvation affect the expression level of ins-1? Assuming that starvation rather increases the release of ins-1, can this trend be independently confirmed by measuring uptake of co-released fluorescent proteins into coelomocytes?

We agree that distinguishing changes in expression from changes in release is important. Previous study suggests that *ins-1* expression is not increased during starvation based on *ins-1p*::GFP::PEST (PMID: 33074105).

To address the reviewer’s suggestion, we performed a coelomocyte uptake assay using a conventional secretion reporter in which INS-1 is fused to full length GFP (*ges-1p::ins-1::GFP*).Coelomocyte fluorescence is significantly lower in starved animals than in fed controls (Supplementary Figure 2A–D). This indicates that starvation does not increase the bulk extracellular availability of INS-1::GFP. On the other hand, we observed the trend of increased signals using neuronal reconstitution of INS-1::GFP11 in the starved condition (Figure 2J), as fluorescence remains readily detectable under both nutritional states (Figure 2G–H). Thus, we do not observe evidence for a starvation-induced reduction in *ins-1* expression.

Taken together, these results suggest that starvation does not increase *ins-1* production or its overall release into the body cavity. Therefore, the change we see in Figure 2 is not explained by higher expression or bulk secretion, but more likely reflects a shift in how INS-1 is routed or delivered to specific neuronal targets under starvation conditions. We revised Discussion as follows to make this point clear:

“Previous work suggests that ins-1 expression is not increased during starvation [56]. Our coelomocyte uptake assay showing that coelomocyte fluorescence is significantly lower in starved animals than in fed controls (S2 Fig), indicates that starvation does not increase bulk extracellular INS-1 availability. In contrast, neuronal split GFP reconstitution signals showed a trend toward increased localization in starved conditions (Fig 2J). These results suggest that starvation does not increase INS-1 production or overall secretion into the body cavity but instead may shift how INS-1 is routed or delivered to specific neuronal targets under starvation conditions.” (Discussion, Page 18, lines 430-437)

4. In Figures 4 and 5, do unc-31, snt-2, egl-21 mutations affect the expression of GFP11-labeled neuropeptides? While the hypothesis that these mutations all negatively regulate the release of NLP-40 is interesting, the interpretation would be more robust if supported by independent evidence, such as coelomocyte uptake assays of co-released fluorescent markers.

As in our response to the reviewer’s Point 3 above, we agree that it is important to distinguish effects on neuropeptide expression from effects on release. We did not directly test the expression of GFP11-labeled neuropeptides because it is difficult to measure the expression using strains based on extrachromosomal arrays. However, at least the expression levels of the co-injection marker under the control of the same promoter as the *ins-1*::GFP11 appeared similar to the control conditions (Fig. 4A–D).

To address the reviewer’s suggestion, we have now performed coelomocyte uptake assays using a conventional secretion reporter in which full-length NLP-40 is fused to GFP (*ges-1p::nlp-40::GFP*). We quantified GFP fluorescence in anterior, midbody, and posterior coelomocytes in wild-type*, unc-31*, *snt-2* and *egl-21* animals. Coelomocyte signal was not significantly different among the four genotypes (Supplementary Figure 3), indicating that overall intestinal expression and bulk extracellular release of NLP-40::GFP are preserved in these mutants.

Taken together, the maintenance of fluorescence in peptide-producing cells and the normal coelomocyte uptake of NLP-40::GFP support the conclusion that the effects observed in Figures 4 and 5 are neither due to a major reduction in neuropeptide expression nor general secretion capacity. We have added Supplementary Figure 3 and updated the Results to make this point explicit:

“Furthermore, we independently assessed secretion by coelomocyte uptake using a intestine-derived NLP-40 tagged with full-length GFP (S3A–E Fig). We quantified GFP fluorescence in anterior, midbody, and posterior coelomocytes in wild-type, *unc-31*, *snt-2,* and *egl-21* animals. Coelomocyte signal was not statistically different among the four genotypes (S3F Fig), indicating that overall intestinal expression and bulk extracellular release of NLP-40::GFP are preserved in these mutants.” (Results, Page 16, lines 384-390)

5. In Figure 5A-F, snt-2 cannot be compared to wild type since it’s carrying glo-4.

We have excluded that from the analysis.

Minor issues:
1. in Line 161 and 290, “neuropeptide signaling” sounds including signaling downstream receptor-mediated events. However, the observations here seem more related to neuropeptide dispersion or extracellular localization. Please revise to avoid confusion.

Thank you for your observation. We have revised the text accordingly:

“On the other hand, animals from the genetic cross of these two strains displayed green punctate signals along the nerve ring, where AFD and AIY contact (Fig 2C), demonstrating that split GFP can visualize neuropeptide released between synaptic partners.” (Results, Page 9, lines 203-205)

2. Figure 3B and E appear unnecessary.

We have removed those figures.

3. Please clarify the second point described in Line 298.

We have clarified the second point as follows:

“Second, this study could not rule out the presence of reconstituted intracellular GFP signals either during synthesis or after endocytosis, and such signals may not respond to regulation as expected. Especially when both GFP1–10::CD4 and GFP11-tagged neuropeptides are expressed in the same cells, GFP complementation could occur intracellularly within the secretory pathway, rather than exclusively at the cell surface. Accordingly, experiments in which GFP fragments are broadly co-expressed (e.g., Fig 1C–E) are interpreted as demonstration of assay feasibility and sensitivity. Nonetheless, our observations support reconstitution after the secretory pathways because GFP signals were predominantly observed along neuronal processes, which are devoid of rough ER markers [59], rather than in perinuclear regions. Especially in the experiments involving anatomically distinct source and target cells (e.g., AFD–AIY), extracellular reconstitution is strongly supported.” (Discussion, Page 20, lines 467-477)
