# Supplementary material for: Neuropeptide visualization using split GFP in live C. elegans
Source: PLoS One. 2026 Aug 19;21(8):e0355191. doi: 10.1371/journal.pone.0355191 (PMC13489428; doi:10.1371/journal.pone.0355191)
Supplement: Peer review history

# Peer review: Decision Letter 0

**DOI:** 10.1371/journal.pone.0355191.r001

**Authors:** Vishwanatha R. A. P. Reddy

24 Sep 2025

Dear Dr. Noma<!--EndFragment-->,

Thank you for submitting your manuscript to PLOS ONE. After careful consideration, we feel that it has merit but does not fully meet PLOS ONE’s publication criteria as it currently stands. Therefore, we invite you to submit a revised version of the manuscript that addresses the points raised during the review process. Please address all the three reviewers comments.

Please submit your revised manuscript by Nov 08 2025 11:59PM. If you will need more time than this to complete your revisions, please reply to this message or contact the journal office at plosone@plos.org. When you're ready to submit your revision, log on to https://www.editorialmanager.com/pone/ and select the 'Submissions Needing Revision' folder to locate your manuscript file.

- A rebuttal letter that responds to each point raised by the academic editor and reviewer(s). You should upload this letter as a separate file labeled 'Response to Reviewers'.
- A marked-up copy of your manuscript that highlights changes made to the original version. You should upload this as a separate file labeled 'Revised Manuscript with Track Changes'.
- An unmarked version of your revised paper without tracked changes. You should upload this as a separate file labeled 'Manuscript'.

If you would like to make changes to your financial disclosure, please include your updated statement in your cover letter. Guidelines for resubmitting your figure files are available below the reviewer comments at the end of this letter.

If applicable, we recommend that you deposit your laboratory protocols in protocols.io to enhance the reproducibility of your results. Protocols.io assigns your protocol its own identifier (DOI) so that it can be cited independently in the future. For instructions see: https://journals.plos.org/plosone/s/submission-guidelines#loc-laboratory-protocols. Additionally, PLOS ONE offers an option for publishing peer-reviewed Lab Protocol articles, which describe protocols hosted on protocols.io. Read more information on sharing protocols at https://plos.org/protocols?utm_medium=editorial-email&utm_source=authorletters&utm_campaign=protocols.

We look forward to receiving your revised manuscript.

Kind regards,

Vishwanatha R. A. P. Reddy

Academic Editor

PLOS ONE

Journal Requirements:

When submitting your revision, we need you to address these additional requirements.

1. Please ensure that your manuscript meets PLOS ONE's style requirements, including those for file naming. The PLOS ONE style templates can be found at

https://journals.plos.org/plosone/s/file?id=wjVg/PLOSOne_formatting_sample_main_body.pdf and

https://journals.plos.org/plosone/s/file?id=ba62/PLOSOne_formatting_sample_title_authors_affiliations.pdf.

2. Thank you for stating in your Funding Statement:

“K.N. received the awards.

This work was supported by MEGMILK SNOW BRAND company and JST FOREST Program, Grant Number JPMJFR 214V.

The funders did not play any role in the study design, data collection and analysis, decision to publish, or preparation of the manuscript.”

Please provide an amended statement that declares *all* the funding or sources of support (whether external or internal to your organization) received during this study, as detailed online in our guide for authors at http://journals.plos.org/plosone/s/submit-now.  Please also include the statement “There was no additional external funding received for this study.” in your updated Funding Statement.

Please include your amended Funding Statement within your cover letter. We will change the online submission form on your behalf.

3. Thank you for stating the following in your manuscript:

“This work was supported by MEGMILK SNOW BRAND company and JST FOREST Program, Grant Number JPMJFR 214V.**”**

We note that you have provided additional information within the Acknowledgements Section that is not currently declared in your Funding Statement. Please note that funding information should not appear in the Acknowledgments section or other areas of your manuscript. We will only publish funding information present in the Funding Statement section of the online submission form.

Please remove any funding-related text from the manuscript and let us know how you would like to update your Funding Statement. Currently, your Funding Statement reads as follows:

“K.N. received the awards

This work was supported by MEGMILK SNOW BRAND company and JST FOREST Program, Grant Number JPMJFR 214V.

The funders did not play any role in the study design, data collection and analysis, decision to publish, or preparation of the manuscript.”

Please include your amended statements within your cover letter; we will change the online submission form on your behalf.

4. We notice that your supplementary tables are included in the manuscript file. Please remove them and upload them with the file type 'Supporting Information'. Please ensure that each Supporting Information file has a legend listed in the manuscript after the references list.

5. If the reviewer comments include a recommendation to cite specific previously published works, please review and evaluate these publications to determine whether they are relevant and should be cited. There is no requirement to cite these works unless the editor has indicated otherwise.

Reviewers' comments:

Reviewer's Responses to Questions

**Comments to the Author**

1. Is the manuscript technically sound, and do the data support the conclusions?

Reviewer #1: Partly

Reviewer #2: Partly

**********

2. Has the statistical analysis been performed appropriately and rigorously?

Reviewer #1: Yes

Reviewer #2: Yes

**********

3. Have the authors made all data underlying the findings in their manuscript fully available?

The PLOS Data policy

Reviewer #1: Yes

Reviewer #2: Yes

**********

4. Is the manuscript presented in an intelligible fashion and written in standard English?

Reviewer #1: Yes

Reviewer #2: Yes

**********

Reviewer #1:

Aleogho and Noma report a novel method for observing neuropeptides and their secretion. They used a split GFP, which allows the observation of neuropeptide binding to a non-endogenous receptor (CD4). They show that tagging two neuropeptides (INS-1 and NLP-40) with the GFP11 fragment doesn’t interfere with their function.

This technique is interesting and promising to better understand how neuropeptides are secreted and interact with receptors.

I do have some questions about the study:

Major:

1. You state in the introduction that the interaction between GFP11 and GFP1-10 is irreversible (p2 line 68), I would agree with that statement. However, this brings some concerns about how it could affect the animals as it means the neuropeptide would bind “forever” to its receptors (here CD4, but in further study, endogenous receptor). It would be interesting to confirm this by doing some dynamic studies or seeing the evolution of GFP with age.

2. Were the behavior assays (defecation, salt conditioning) executed with the expression of GFP1-10::CD40 or just the tagging (GFP11) of the neuropeptides? In the legend and the text, it only talks about neuropeptide tagging. It is essential to test it with GFP1-10::CD40 expression, also to show that having the neuropeptides being taken away from their endogenous receptors doesn’t interfere with the function of those neuropeptides, which could explain some of the rescue results (aka no rescue).

3. In the first part of the study, GFP1-10::CD40 and INS-1::GFP11 are expressed in the same cells (Fig1). It would be important to indicate that the signal observed could be due to their interaction in the same cells, including intracellular binding.

4. Fig 2: The image H (starved) looks like the GFP signal is less diffused than in G(fed). Is this representative? Would it be supported by the known role of INS-1 during starvation? Could the difference be tested (number, size, and/or intensity of the puncta observed)?

5. Fig 3 G: Can you explain/ give hypotheses of why the rescue is better in the glo-4 background?

6. Fig 3G: Can you explain and give more details and hypotheses for the lack of rescue in the defecation assay? Is it due to the expression go NPL-40 in the gut, and maybe neuron rescue is also necessary for this behavior? Or could the GFP interfere with P3 binding to its AEX-2 receptor? Could the P3 and P4 tagging be done as a knock-in instead of overexpression to avoid mosaic expression?

7. Fig 4: Some of the results are quite variable, which may be due to the mosaicism of extrachromosomal lines. It often happens that the GFP expression of extrachromosomal arrays changes after crossing. It would be better to do those experiments in integrated lines, which would have a more stable expression. The KI of GFP11 would be a great way to control the expression, too, with integrated CD40.

8. Those results (Fig4) showing an increase in fluorescence are really interesting and are worth exploring further. Does the signal look more diffused? Less localized at the synapse, maybe? I guess with CD4 being a non-endogenous receptor means it is not localized at the synapses, and could explain the diffusion of the signal. This is where it would be good to expand the discussion on this part, especially given the results from Wang et al (2013). They demonstrated that in the snt-2 mutant, NPL-40 is less secreted due to a lower intensity of YFP from the NLP-40 transgenic reporter in the coelomocytes.

Minor:

1. Can you explain why the glo-4 background could not be isolated in some strains?

2. Line 225, I assumed you used the non-transgenic siblings as a control, or did you isolate a line from non-transgenic siblings before evaluation? Could you clarify?

3. In Fig 2F, a picture of the ventral nerve cord expression is missing. You state in the text that you can observe GFP in the nerve cord, but you do not support that claim with an image in the figure. Would it be possible to add one?

4. Recently, the use of rol-6 as a co-injection marker has been shown to affect synaptic phenotypes in male neurons (Jin et al (2019, 10.1371/journal.pone.0224533). Even though this study focuses on males, it would be good to talk about this caveat in the results, so people can choose a different co-injection marker if they wish.

Reviewer #2:

In this study, Aleogho et al. applied the split GFP system for in vivo visualization of neuropeptide release in C. elegans, focusing on INS-1 and NLP-40. The advantage of this approach is that tagging neuropeptide with GFP11 minimizes the functional disruption of neuropeptide compared to full-length fluorescent tags. It allows detection of GFP11-tagged neuropeptide at sites where GFP1-10 is expressed. The paper is straightforward and easy to follow. The observation that INS-1 and NLP-40 secretion from AFD and intestine was not inhibited in unc-31, snt-2, or egl-21 is unexpected and interesting, and possibly worth further exploring.

I have a few concerns:

1. Methods: The manuscript does not provide sufficient information on construct design and experimental procedures. For instance, the DNA templates used for GFP11 knock-ins, the signal sequences used for GFP1-10 targeting, and primer sequences for cloning are not described. In the chemotaxis assay, the author indicated n = 7 but it remains unclear if these assays were 7 technical replicates or from different biological replication. It remains unclear by checking the source data. It is also unclear if the displayed images were the maximum projections of z-stacks or taken from a single focal plane.

2. References: A recent publication by Kurashina, using split GFP and wrmScarlet systems to label endogenous presynaptic protein, was not cited.

3. Neurons: Could authors explain why they choose AFD neurons, in which ins-1 does not seem to be endogenously expressed? Would this, and intestinal overexpression, underlie the unexpected secretion of two neuropeptides in different mutants? Would it be better if the ins-1::GFP11 knock-in strain or different neurons such as AIA were used?

4. It is worth discussing how the method could be used to map neuropeptide signal transduction in physiologically relevant contexts.

Reviewer #3:

Aleogho et al. developed a methodology to visualize the released neuropeptides using a split GFP system and quantified their release under different conditions, including fed vs. starved states and in various mutant backgrounds.

Main criticism:

Given the current limitation of methodologies to monitor neuropeptide release, this work holds substantial potential. Nonetheless, there are some unexpected findings, and it is not clear if the methodology is sufficiently benchmarked using the reported examples:

NLP-40 and INS-1 are not affected by mutations that should influence neuropeptide release (unc-31, snt-2). This may be a specific feature of these two neuropeptides, that are released from a large tissue, i.e. the intestine, in addition to other cells. The authors should provide an example of a purely neuronally released neuropeptide, possibly from the FMRFamide class, that is known to be affected by unc-31 for its release (several examples can be found in the literature, e.g. NLP-21 or FLP-1). To thus see if this can be recapitulated by their assay, i.e. no release in such a mutant and thus no signal.

Generally, the authors should also probe the limits of their approach by choosing a neuropeptide that is expressed in very few cells and at low expression level, to see if this can nonetheless be picked up by this approach. Using neuropeptides with high expression level in large cells, that will inevitably produce high amounts of secreted peptide, are probably more likely to produce detectable signal fom GFP reconstitution.

In addition, several key statements in the manuscripts are not sufficiently supported by robust evidence. I outline major and minor concerns below.

Major issues:

1. In Figure 1, CD4-GFP-1-10 and GFP11 are both expressed pan-neuronally. In Figure 2G-J, CD4-GFP-1-10 and GFP11 are supposed to be both expressed in many neurons, given the widespread expression of ins-1. Is GFP reassembled really on the extracellular surface of the cells, rather than inside ER?

2. Many statements are based on results without significant differences. e.g. Figures 2J, 3I, 4L. In such cases, the corresponding interpretations should be reconsidered or removed from the main conclusions.

3. Regarding Figure 2J, does starvation affect the expression level of ins-1? Assuming that starvation rather increases the release of ins-1, can this trend be independently confirmed by measuring uptake of co-released fluorescent proteins into coelomocytes?

4. In Figures 4 and 5, do unc-31, snt-2, egl-21 mutations affect the expression of GFP11-labeled neuropeptides? While the hypothesis that these mutations all negatively regulate the release of NLP-40 is interesting, the interpretation would be more robust if supported by independent evidence, such as coelomocyte uptake assays of co-released fluorescent markers.

5. In Figure 5A-F, snt-2 cannot be compared to wild type since it’s carrying glo-4.

Minor issues:

1. in Line 161 and 290, “neuropeptide signaling” sounds including signaling downstream receptor-mediated events. However, the observations here seem more related to neuropeptide dispersion or extracellular localization. Please revise to avoid confusion.

2. Figure 3B and E appear unnecessary.

3. Please clarify the second point described in Line 298.

**********

what does this mean?). If published, this will include your full peer review and any attached files.

If you choose “no”, your identity will remain anonymous but your review may still be made public.

**Do you want your identity to be public for this peer review?**  For information about this choice, including consent withdrawal, please see our Privacy Policy

Reviewer #1: No

Reviewer #2: No

**********

[NOTE: If reviewer comments were submitted as an attachment file, they will be attached to this email and accessible via the submission site. Please log into your account, locate the manuscript record, and check for the action link "View Attachments". If this link does not appear, there are no attachment files.]

While revising your submission, please upload your figure files to the Preflight Analysis and Conversion Engine (PACE) digital diagnostic tool, https://pacev2.apexcovantage.com/. PACE helps ensure that figures meet PLOS requirements. To use PACE, you must first register as a user. Registration is free. Then, login and navigate to the UPLOAD tab, where you will find detailed instructions on how to use the tool. If you encounter any issues or have any questions when using PACE, please email PLOS at figures@plos.org. Please note that Supporting Information files do not need this step.

# Author comment: Author response to Decision Letter 1

**DOI:** 10.1371/journal.pone.0355191.r002

8 Feb 2026

Rebuttal letter for “Neuropeptide visualization using split GFP in live C. elegans” by Aleogho et al.

February 8, 2026

We appreciate the constructive feedback from the editor and reviewers and have substantially revised the manuscript based on the comments. Our responses are shown in blue, while the original comments are in black. The cited portions of the main text are enclosed in quotation marks and underlined. All changes in the manuscript text were tracked. Some figure panels were replaced and new figures were added. Please note that in our responses below, any of such references refer to the revised manuscript.

We are glad the reviewers found our study interesting and offered constructive comments. As the reviewers suggested, we added new experiments [Reviewer #1 (points 1 and 2) and Reviewer #3 (points 3 and 4)] and clarified some points in the Introduction, Results, and Discussion.

The following new data were added:

• Supplementary Figure 1

• Supplementary Figure 2

• Supplementary Figure 3

The following figures were replaced:

• Figure 1J

• Figure 3E

• Figure 3G

Editor’s Comments:

Journal Requirements:

When submitting your revision, we need you to address these additional requirements.

1. Please ensure that your manuscript meets PLOS ONE's style requirements, including those for file naming. The PLOS ONE style templates can be found at

https://journals.plos.org/plosone/s/file?id=wjVg/PLOSOne_formatting_sample_main_body.pdf and

https://journals.plos.org/plosone/s/file?id=ba62/PLOSOne_formatting_sample_title_authors_affiliations.pdf.

We have carefully formatted the manuscript according to PLOS ONE’s style requirements. We effected the following changes:

• Converted the manuscript to double-space paragraph format

• Moved figure captions to appear after the paragraphs in which they’re first mentioned

• Named files appropriately

2. Thank you for stating in your Funding Statement:

“K.N. received the awards.

This work was supported by MEGMILK SNOW BRAND company and JST FOREST Program, Grant Number JPMJFR 214V.

The funders did not play any role in the study design, data collection and analysis, decision to publish, or preparation of the manuscript.”

Please provide an amended statement that declares *all* the funding or sources of support (whether external or internal to your organization) received during this study, as detailed online in our guide for authors at http://journals.plos.org/plosone/s/submit-now. Please also include the statement “There was no additional external funding received for this study.” in your updated Funding Statement.

Please include your amended Funding Statement within your cover letter. We will change the online submission form on your behalf.

Thank you. We have provided an amended Funding Statement on the online submission system, cover letter, and manuscript.

3. Thank you for stating the following in your manuscript:

“This work was supported by MEGMILK SNOW BRAND company and JST FOREST Program, Grant Number JPMJFR 214V.”

We note that you have provided additional information within the Acknowledgements Section that is not currently declared in your Funding Statement. Please note that funding information should not appear in the Acknowledgments section or other areas of your manuscript. We will only publish funding information present in the Funding Statement section of the online submission form.

Please remove any funding-related text from the manuscript and let us know how you would like to update your Funding Statement. Currently, your Funding Statement reads as follows:

“K.N. received the awards

This work was supported by MEGMILK SNOW BRAND company and JST FOREST Program, Grant Number JPMJFR 214V.

The funders did not play any role in the study design, data collection and analysis, decision to publish, or preparation of the manuscript.”

Please include your amended statements within your cover letter; we will change the online submission form on your behalf.

We apologize for the oversight. We have removed the funding-related text from the Acknowledgements sections and added it to the amended Funding section on the manuscript, online Funding Statement, and cover letter.

4. We notice that your supplementary tables are included in the manuscript file. Please remove them and upload them with the file type 'Supporting Information'. Please ensure that each Supporting Information file has a legend listed in the manuscript after the references list.

We have removed the tables from the manuscript and uploaded them with the ‘Supporting Information’ file type.

5. If the reviewer comments include a recommendation to cite specific previously published works, please review and evaluate these publications to determine whether they are relevant and should be cited. There is no requirement to cite these works unless the editor has indicated otherwise.

We note this and thank the editor. After evaluation, we included the reference Kurashina et al, 2025 suggested by Reviewer #2 (point 2). (Ref. 36. Page 3, line 71).

Reviewers' comments:

Reviewer's Responses to Questions

Comments to the Author

1. Is the manuscript technically sound, and do the data support the conclusions?

The manuscript must describe a technically sound piece of scientific research with data that supports the conclusions. Experiments must have been conducted rigorously, with appropriate controls, replication, and sample sizes. The conclusions must be drawn appropriately based on the data presented.

Reviewer #1: Partly

Reviewer #2: Partly

________________________________________

2. Has the statistical analysis been performed appropriately and rigorously?

Reviewer #1: Yes

Reviewer #2: Yes

________________________________________

3. Have the authors made all data underlying the findings in their manuscript fully available?

The PLOS Data policy requires authors to make all data underlying the findings described in their manuscript fully available without restriction, with rare exception (please refer to the Data Availability Statement in the manuscript PDF file). The data should be provided as part of the manuscript or its supporting information, or deposited to a public repository. For example, in addition to summary statistics, the data points behind means, medians and variance measures should be available. If there are restrictions on publicly sharing data—e.g. participant privacy or use of data from a third party—those must be specified.

Reviewer #1: Yes

Reviewer #2: Yes

________________________________________

4. Is the manuscript presented in an intelligible fashion and written in standard English?

PLOS ONE does not copyedit accepted manuscripts, so the language in submitted articles must be clear, correct, and unambiguous. Any typographical or grammatical errors should be corrected at revision, so please note any specific errors here.

Reviewer #1: Yes

Reviewer #2: Yes

________________________________________

5. Review Comments to the Author

Please use the space provided to explain your answers to the questions above. You may also include additional comments for the author, including concerns about dual publication, research ethics, or publication ethics. (Please upload your review as an attachment if it exceeds 20,000 characters)

Reviewer #1:

Aleogho and Noma report a novel method for observing neuropeptides and their secretion. They used a split GFP, which allows the observation of neuropeptide binding to a non-endogenous receptor (CD4). They show that tagging two neuropeptides (INS-1 and NLP-40) with the GFP11 fragment doesn’t interfere with their function.

This technique is interesting and promising to better understand how neuropeptides are secreted and interact with receptors.

We are glad that the reviewer finds this work interesting and promising.

I do have some questions about the study:

Major:

1. You state in the introduction that the interaction between GFP11 and GFP1-10 is irreversible (p2 line 68), I would agree with that statement. However, this brings some concerns about how it could affect the animals as it means the neuropeptide would bind “forever” to its receptors (here CD4, but in further study, endogenous receptor). It would be interesting to confirm this by doing some dynamic studies or seeing the evolution of GFP with age.

We thank the reviewer for this comment and agree that the stable interaction between GFP11 and GFP1–10 may perturb the original signaling from a tagged neuropeptide to its receptor. To reduce the distortion due to irreversible binding, we may be able to use a split fluorescent tag that allows reversible binding (PMID: 31249300). It was not our first choice because we would sacrifice the intensity. Further optimization will be necessary in the future. We added discussion regarding these matters as shown below:

“Another limitation of the split GFP is the irreversible binding of GFP11 and GFP1-10, which might perturb endogenous signaling dynamics. Future optimization may address this limitation through the use of alternative split fluorescent systems with reversible complementation.” (Discussion, Page 21, lines 487-490).

Furthermore, to directly address the reviewer’s concern regarding signal dynamics, we analyzed the change of GFP fluorescence with age (Supplementary Figure 1). We observed a progressive increase in GFP signal from Day 1 to Day 5, consistent with cumulative detection of interaction events over time rather than sustained receptor engagement. This observation is expected for irreversible GFP complementation. The main text was revised accordingly:

“Since the binding between INS-1::GFP11 and GFP1-10::CD4 is thought to be irreversible, the observed signals likely reflect the accumulation of past secretion events. To assess whether split GFP signal accumulates over time, we quantified fluorescence intensity of ins-1::GFP11 KI and pan-neuronal GFP1-10::CD4 at different adult ages (S1A–D Fig). GFP signal increased progressively from Day 1 to Day 5 (S1E Fig), consistent with integration of reconstituted GFP signals.” (Results, Page 7, line 167 – Page 8, line 172)

2. Were the behavior assays (defecation, salt conditioning) executed with the expression of GFP1-10::CD40 or just the tagging (GFP11) of the neuropeptides? In the legend and the text, it only talks about neuropeptide tagging. It is essential to test it with GFP1-10::CD40 expression, also to show that having the neuropeptides being taken away from their endogenous receptors doesn’t interfere with the function of those neuropeptides, which could explain some of the rescue results (aka no rescue).

We thank the reviewer for raising this concern. The reviewer is correct; in the original manuscript, we executed behavioral assays with strains expressing just the tagging (GFP11) of the neuropeptides to address if tagging itself perturb the functionality. Based on the reviewer’s suggestion, we conducted an additional experiment to see whether co-expression of GFP1–10::CD4 and ins-1::GFP11 affects behavioral outputs by performing NaCl chemotaxis assays. As shown in Figure 1J, animals co-expressing ins-1::GFP11 and GFP1–10::CD4 displayed chemotaxis indices that were not significantly different from wild-type controls in the Naïve or Mock-conditioned groups and showed the expected reduction in chemotaxis following starvation conditioning with NaCl. These results indicate that co-expression of ins-1::GFP11 and GFP1–10::CD4 does not impair NaCl chemotaxis or learning and therefore does not measurably perturb this behavioral output. We have clarified these points in the Results section to explicitly state which behavioral assay was performed in the presence of GFP1–10::CD4:

“The ins-1::GFP11 KI animals behaved similarly to the wild type across all three groups (Fig 1J), indicating normal function of GFP11-tagged INS-1. Moreover, co-expression of GFP1–10::CD4 with ins-1::GFP did not significantly alter NaCl chemotaxis in the Naïve or Mock-conditioned groups and only slightly prevented learning-induced changes in chemotaxis index following NaCl-conditioning (Fig 1J).” (Results, Page 8, lines 180-184)

For the defecation motor program assay, we did not repeat the experiment using GFP1–10::CD4–expressing strains. This decision was based on the consideration that GFP11 tagging likely interfered with the function and/or proper cleavage of P3 peptide (Figure 3G. We discussed further in Major point 6). We independently repeated the original defecation assay with an increased sample size to clarify the initial result. This repeat experiment reproduced the original trend (Figure 3G), supporting the robustness of the conclusion. Given these considerations, we chose not to pursue additional defecation assays with GFP1–10::CD4 strains.

3. In the first part of the study, GFP1-10::CD40 and INS-1::GFP11 are expressed in the same cells (Fig1). It would be important to indicate that the signal observed could be due to their interaction in the same cells, including intracellular binding.

We thank the reviewer for raising that point and have indicated the limitation in the Results section as shown below:

“Because the same neurons might express both GFP1–10::CD4 and ins-1::GFP11 in both configurations in Fig 1C–E, we could not exclude the possibility that the reconstituted GFP signals might originate from intracellular compartments such as ER, Golgi, and endosomes in addition to potential extracellular interactions.” (Page 7, lines 163-166)

4. Fig 2: The image H (starved) looks like the GFP signal is less diffused than in G(fed). Is this representative? Would it be supported by the known role of INS-1 during starvation? Could the difference be tested (number, size, and/or intensity of the puncta observed)?

We thank the reviewer for this comment. Figure 2G (fed) and H (starved) show representative examples of intestine-derived INS-1::GFP11 signal. However, across animals, we did not observe a consistent starvation-dependent change in the overall distribution or appearance of the reconstituted GFP signal. Although, there was a trend of increase, quantification of signal intensity (Fig. 2J) showed no significant difference between fed and starved conditions.

We interpret the apparent difference in signal “diffuseness” between the individual images in Figure 2G and H as falling within normal biological and imaging variability, rather than reflecting a robust starvation-dependent change in INS-1 localization. Because no consistent qualitative difference was observed across animals, we did not further quantify puncta number, size, or intensity, but we mentioned this observation in the Results as follows:

“Compared to well-fed animals, animals starved for 3 hours exhibited a trend of increase in reconstituted GFP signal along the nerve ring, while we did not see any consistent changes in the localization patterns (Fig 2G–J). ” (Page 11, lines 246-248).

5. Fig 3 G: Can you explain/ give hypotheses of why the rescue is better in the glo-4 background?

We are not sure, but there could be genetic interaction between the glo-4 and nlp-40 mutant backgrounds given that both are expressed in the intestine. For simplification and clarity, we removed the glo-4 data and tested more samples of the wild type to validate the previous data. Please see the new Figure 3G. We have also edited the text accordingly.

6. Fig 3G: Can you explain and give more details and hypotheses for the lack of rescue in the defecation assay? Is it due to the expression go NPL-40 in the gut, and maybe neuron rescue is also necessary for this behavior? Or could the GFP interfere with P3 binding to its AEX-2 receptor? Could the P3 and P4 tagging be done as a knock-in instead of overexpression to avoid mosaic expression?

We thank the reviewer for these thoughtful suggestions. We note that in the previous paper by Wang et al (PMID: 23583549), the expulsion defect of nlp-40 mutants was fully rescued by overexpression of nlp-40. However, in Figure 3G of our study, overexpression of intestinal nlp-40(P3)::GFP11 or nlp-40(P4)::GFP11 in the nlp-40 mutant background does not robustly restore expulsion frequency to wild-type levels.

In our nlp-40(P3)::GFP11 and nlp-40(P4)::GFP11 constructs, the full nlp-40 precursor

Attachment Submitted filename: Response to Reviewers_ver6.docx

# Peer review: Decision Letter 1

**DOI:** 10.1371/journal.pone.0355191.r003

**Authors:** Vishwanatha R. A. P. Reddy

8 Apr 2026

Dear Dr. Noma,

Thank you for submitting your manuscript to PLOS ONE. After careful consideration, we feel that it has merit but does not fully meet PLOS ONE’s publication criteria as it currently stands. Therefore, we invite you to submit a revised version of the manuscript that addresses the points raised during the review process.

Please submit your revised manuscript by May 23 2026 11:59PM. If you will need more time than this to complete your revisions, please reply to this message or contact the journal office at plosone@plos.org. When you're ready to submit your revision, log on to https://www.editorialmanager.com/pone/ and select the 'Submissions Needing Revision' folder to locate your manuscript file.

- A letter that responds to each point raised by the academic editor and reviewer(s). You should upload this letter as a separate file labeled 'Response to Reviewers'.
- A marked-up copy of your manuscript that highlights changes made to the original version. You should upload this as a separate file labeled 'Revised Manuscript with Track Changes'.
- An unmarked version of your revised paper without tracked changes. You should upload this as a separate file labeled 'Manuscript'.

If you would like to make changes to your financial disclosure, please include your updated statement in your cover letter. Guidelines for resubmitting your figure files are available below the reviewer comments at the end of this letter.

If applicable, we recommend that you deposit your laboratory protocols in protocols.io to enhance the reproducibility of your results. Protocols.io assigns your protocol its own identifier (DOI) so that it can be cited independently in the future. For instructions see: https://journals.plos.org/plosone/s/submission-guidelines#loc-laboratory-protocols. Additionally, PLOS ONE offers an option for publishing peer-reviewed Lab Protocol articles, which describe protocols hosted on protocols.io. Read more information on sharing protocols at https://plos.org/protocols?utm_medium=editorial-email&utm_source=authorletters&utm_campaign=protocols.

As the corresponding author, your ORCID iD is verified in the submission system and will appear in the published article. PLOS supports the use of ORCID, and we encourage all coauthors to register for an ORCID iD and use it as well. Please encourage your coauthors to verify their ORCID iD within the submission system before final acceptance, as unverified ORCID iDs will not appear in the published article. *Only*  the individual author can complete the verification step; PLOS staff *cannot*  verify ORCID iDs on behalf of authors.

We look forward to receiving your revised manuscript.

Kind regards,

Vishwanatha R. A. P. Reddy

Academic Editor

PLOS One

Journal Requirements:

If the reviewer comments include a recommendation to cite specific previously published works, please review and evaluate these publications to determine whether they are relevant and should be cited. There is no requirement to cite these works unless the editor has indicated otherwise.

Please review your reference list to ensure that it is complete and correct. If you have cited papers that have been retracted, please include the rationale for doing so in the manuscript text, or remove these references and replace them with relevant current references. Any changes to the reference list should be mentioned in the rebuttal letter that accompanies your revised manuscript. If you need to cite a retracted article, indicate the article’s retracted status in the References list and also include a citation and full reference for the retraction notice.

[Note: HTML markup is below. Please do not edit.]

Reviewers' comments:

Reviewer's Responses to Questions

**Comments to the Author**

Reviewer #2: (No Response)

Reviewer #3: (No Response)

Reviewer #4: All comments have been addressed

**********

2. Is the manuscript technically sound, and do the data support the conclusions?

Reviewer #2: Partly

Reviewer #3: Yes

Reviewer #4: Yes

**********

3. Has the statistical analysis been performed appropriately and rigorously?

Reviewer #2: Yes

Reviewer #3: Yes

Reviewer #4: Yes

**********

4. Have the authors made all data underlying the findings in their manuscript fully available?

The PLOS Data policy

Reviewer #2: Yes

Reviewer #3: Yes

Reviewer #4: Yes

**********

5. Is the manuscript presented in an intelligible fashion and written in standard English?

Reviewer #2: Yes

Reviewer #3: Yes

Reviewer #4: Yes

**********

Reviewer #2: I raised four concerns in the initial evaluation. The authors have sufficiently addressed points 1, 2, and 4.

Regarding point 3, I appreciate the clarification on the choice of AFD/AIY promoters. However, my concern was about if the observed unc-31-independent secretion could result from ins-1 overexpression and ectopic expression, and if altered cellular context or overexpression could potentially bypass canonical dense-core vesicle machinery. In particular, if the unc-31 mutants used retain partial activity (as discussed by the authors, 456-457), may overexpression lead to the observed phenotype. I would recommend that the authors discuss alternative possibilities and clarify if unc-31-independent secretion is context-dependent, even though it would be ideal if additional experiments, such as single copy knock-in and AIA expression, were performed.

Reviewer #3: In this work, Aleogho et al. describe a split GFP approach aimed at labeling tissues with access to neuropeptides released from either neurons or the intestine. They show that they could detect fluorescence on the surface of cells that arises specifically from reconstitution of GFP by tagged peptides. The data supporting their conclusion are clear and well presented, and the study convincingly demonstrates that peptides released from specific cells can access the surfaces of other nearby or more distant cells and that this access occurs via a mechanism that likely involves their diffusion within the extracellular fluid following their release. The advantages and limitations of this study in detecting neurosecretion and in quantifying neuropeptide release efficacy were demonstrated experimentally and well described.

Comments:

1) The authors focus on interactions of peptides with cells in the nerve ring and show nice images and quantification that support these interactions. They also mention interactions of peptides with neurons in the ventral nerve cord and they show some images where the anterior nerve cord can be seen (e.g. Fig 2f). It would strengthen the paper if the authors could add additional images of the nerve cord. These images would be helpful because each soma and process can be seen clearly in the same plane. Presumably, NLP-40 from the intestine should have access to these neurons. For this reason, it may be that quantification of fluorescence signal in the ventral nerve cord would be more accurate (and sensitive to changes) than in the nerve ring (error bars of nerve ring fluorescence throughout are quite large—e.g. Fig 4). Although adding images and analysis of the ventral nerve cord could strengthen the paper, adding these images/experiments may not be necessary for publication of this study.

2) Figure 4: The authors report showing no changes in fluorescence of neuropeptides expressed from the intestine in egl-21 or unc-31 mutants. One explanation for the unc-31 observation is that unc-31 is not likely to regulate neuropeptide release from the intestine (since it is expressed there at low levels and unc-31 mutants do not have defecation defects), and a possible explanation for the egl-21 observation is that defects in neuropeptide processing may not be expected to impact their release. Another potential explanation for lack of changes seen in release mutants could be that if the GFP1-10::CD4 is expressed at limiting levels, these receptors could become saturated with even when neuropeptide secretion is compromised, making changes in secretion difficult to detect. These possibilities could be added to the Discussion.

Reviewer #4: (No Response)

**********

what does this mean?). If published, this will include your full peer review and any attached files.

If you choose “no”, your identity will remain anonymous but your review may still be made public.

**Do you want your identity to be public for this peer review?**  For information about this choice, including consent withdrawal, please see our Privacy Policy

Reviewer #2: No

Reviewer #3: No

Reviewer #4: No

**********

[NOTE: If reviewer comments were submitted as an attachment file, they will be attached to this email and accessible via the submission site. Please log into your account, locate the manuscript record, and check for the action link "View Attachments". If this link does not appear, there are no attachment files.]

To ensure your figures meet our technical requirements, please review our figure guidelines: https://journals.plos.org/plosone/s/figures

You may also use PLOS’s free figure tool, NAAS, to help you prepare publication quality figures: https://journals.plos.org/plosone/s/figures#loc-tools-for-figure-preparation.

NAAS will assess whether your figures meet our technical requirements by comparing each figure against our figure specifications.

Attachment Submitted filename: Reviewer Comments_PLosOne.pdf

# Author comment: Author response to Decision Letter 2

**DOI:** 10.1371/journal.pone.0355191.r004

21 Jun 2026

Rebuttal letter for “Neuropeptide visualization using split GFP in live C. elegans” by Aleogho et al., 2nd round

June 22, 2026

We appreciate the constructive feedback from the reviewers. We have revised the main texts based on the comments, but did not add any additional data in this round of revision. Our responses are shown in blue, while the original comments are in black. The cited portions of the main text are enclosed in quotation marks. All changes in the manuscript text were tracked in the tracked version.

Reviewers' comments:

Reviewer #2: I raised four concerns in the initial evaluation. The authors have sufficiently addressed points 1, 2, and 4.

Regarding point 3, I appreciate the clarification on the choice of AFD/AIY promoters. However, my concern was about if the observed unc-31-independent secretion could result from ins-1 overexpression and ectopic expression, and if altered cellular context or overexpression could potentially bypass canonical dense-core vesicle machinery. In particular, if the unc-31 mutants used retain partial activity (as discussed by the authors, 456-457), may overexpression lead to the observed phenotype. I would recommend that the authors discuss alternative possibilities and clarify if unc-31-independent secretion is context-dependent, even though it would be ideal if additional experiments, such as single copy knock-in and AIA expression, were performed.

We appreciate that the reviewer raised an important point. We did not use ins-1 knock-in animals for mutant analyses for two reasons. First, ins-1 is close to egl-21 and unc-31 on the same chromosome (IV, ins-1, 4.59; egl-21, 4.60; unc-31, 6.32). Second, ins-1 knock-in produced weaker signals than ins-1 overexpression strains. Therefore, instead of doing additional experiments using knock-in strains, we added the following discussion:

Discussion, page 20, Line 479-481

“Another limitation is that the mutant analyses relied on exogenously overexpressed INS-1 and NLP-40 using the extrachromosomal arrays, which may mask the endogenous regulation of these neuropeptides.”

Reviewer #3: In this work, Aleogho et al. describe a split GFP approach aimed at labeling tissues with access to neuropeptides released from either neurons or the intestine. They show that they could detect fluorescence on the surface of cells that arises specifically from reconstitution of GFP by tagged peptides. The data supporting their conclusion are clear and well presented, and the study convincingly demonstrates that peptides released from specific cells can access the surfaces of other nearby or more distant cells and that this access occurs via a mechanism that likely involves their diffusion within the extracellular fluid following their release. The advantages and limitations of this study in detecting neurosecretion and in quantifying neuropeptide release efficacy were demonstrated experimentally and well described.

Comments:

1) The authors focus on interactions of peptides with cells in the nerve ring and show nice images and quantification that support these interactions. They also mention interactions of peptides with neurons in the ventral nerve cord and they show some images where the anterior nerve cord can be seen (e.g. Fig 2f). It would strengthen the paper if the authors could add additional images of the nerve cord. These images would be helpful because each soma and process can be seen clearly in the same plane. Presumably, NLP-40 from the intestine should have access to these neurons. For this reason, it may be that quantification of fluorescence signal in the ventral nerve cord would be more accurate (and sensitive to changes) than in the nerve ring (error bars of nerve ring fluorescence throughout are quite large—e.g. Fig 4). Although adding images and analysis of the ventral nerve cord could strengthen the paper, adding these images/experiments may not be necessary for publication of this study.

We appreciate the reviewer’s comments and suggestions. However, we decided not to include additional images. We were able to observe the nerve cord even when we focused on the nerve ring region and found similar variability and trends in both regions. Moreover, we could detect NLP-40 signals even in the anterior nerve cord and nerve ring. Therefore, we do not believe that the issue is the distance from the site of NLP-40 secretion. Instead, the major limitation is likely the use of extrachromosomal arrays. Regarding that point, we added the following sentence in Discussion (see Reviewer 2’s comment as well):

Discussion, page 20, Line 479-481

“Another limitation is that the mutant analyses relied on exogenously overexpressed INS-1 and NLP-40 using the extrachromosomal arrays, which may mask the endogenous regulation of these neuropeptides.”

2) Figure 4: The authors report showing no changes in fluorescence of neuropeptides expressed from the intestine in egl-21 or unc-31 mutants. One explanation for the unc-31 observation is that unc-31 is not likely to regulate neuropeptide release from the intestine (since it is expressed there at low levels and unc-31 mutants do not have defecation defects), and a possible explanation for the egl-21 observation is that defects in neuropeptide processing may not be expected to impact their release. Another potential explanation for lack of changes seen in release mutants could be that if the GFP1-10::CD4 is expressed at limiting levels, these receptors could become saturated with even when neuropeptide secretion is compromised, making changes in secretion difficult to detect. These possibilities could be added to the Discussion.

Thank you very much for clarifying the different possibilities. We agree that unc-31 is not strongly expressed in the intestine and added the following sentence in Discussion. We did not mention the absence of defecation defect because unc-31 is reported to have a defecation defect (Speese et al., J. Neurosci, 2007, PMID: 17553987).

Discussion, page 19, Line 454-456

“For instance, the lack of unc-31-dependency of the intestine-derived INS-1 and NLP-40 may be explained by weak or no expression of unc-31 in the intestine [51].”

The relationship between neuropeptide processing and secretion is an important point. We added the following discussion about egl-21, based on the suggestion. Thank you for pointing it out.

Discussion, page 19, Line 461-462

“Regarding the egl-21 mutant, neuropeptide processing by EGL-21 might not be required for neuropeptide secretion.”

We also added the following sentence regarding the saturation of GFP1-10 as suggested.

Discussion, page 20, Line 479-481

“In addition, the limited amount of GFP1-10::CD4 may have become saturated, thereby hindering the detection of changes in neuropeptide secretion in the mutant backgrounds.”

Attachment Submitted filename: Response to Reviewers2_ver2.pdf

# Editor report: Decision Letter 2

**DOI:** 10.1371/journal.pone.0355191.r005

**Authors:** Vishwanatha R. A. P. Reddy

20 Jul 2026

Neuropeptide visualization using split GFP in live C. elegans

PONE-D-25-43882R2

Dear Dr. Noma,

We’re pleased to inform you that your manuscript has been judged scientifically suitable for publication and will be formally accepted for publication once it meets all outstanding technical requirements.

Within one week, you’ll receive an e-mail detailing the required amendments. When these have been addressed, you’ll receive a formal acceptance letter and your manuscript will be scheduled for publication.

An invoice will be generated when your article is formally accepted. Please note, if your institution has a publishing partnership with PLOS and your article meets the relevant criteria, all or part of your publication costs will be covered. Please make sure your user information is up-to-date by logging into Editorial Manager at Editorial Manager® and clicking the ‘Update My Information' link at the top of the page. For questions related to billing, please contact billing support.

If your institution or institutions have a press office, please notify them about your upcoming paper to help maximize its impact. If they’ll be preparing press materials, please inform our press team as soon as possible -- no later than 48 hours after receiving the formal acceptance. Your manuscript will remain under strict press embargo until 2 pm Eastern Time on the date of publication. For more information, please contact onepress@plos.org.

Kind regards,

Vishwanatha R. A. P. Reddy

Academic Editor

PLOS One

Additional Editor Comments (optional):

Congratulations. Well done.

Reviewers' comments:

# Editor report: Acceptance letter

**DOI:** 10.1371/journal.pone.0355191.r006

**Authors:** Vishwanatha R. A. P. Reddy

PONE-D-25-43882R2

PLOS One

Dear Dr. Noma,

I'm pleased to inform you that your manuscript has been deemed suitable for publication in PLOS One. Congratulations! Your manuscript is now being handed over to our production team.

At this stage, our production department will prepare your paper for publication. This includes ensuring the following:

* All references, tables, and figures are properly cited

* All relevant supporting information is included in the manuscript submission,

* There are no issues that prevent the paper from being properly typeset

You will receive further instructions from the production team, including instructions on how to review your proof when it is ready. Please keep in mind that we are working through a large volume of accepted articles, so please give us a few days to review your paper and let you know the next and final steps.

Lastly, if your institution or institutions have a press office, please let them know about your upcoming paper now to help maximize its impact. If they'll be preparing press materials, please inform our press team within the next 48 hours. Your manuscript will remain under strict press embargo until 2 pm Eastern Time on the date of publication. For more information, please contact onepress@plos.org.

You will receive an invoice from PLOS for your publication fee after your manuscript has reached the completed accept phase. If you receive an email requesting payment before acceptance or for any other service, this may be a phishing scheme. Learn how to identify phishing emails and protect your accounts at https://explore.plos.org/phishing.

If we can help with anything else, please email us at customercare@plos.org.

Thank you for submitting your work to PLOS ONE and supporting open access.

Kind regards,

PLOS ONE Editorial Office Staff

on behalf of

Dr. Vishwanatha R. A. P. Reddy

Academic Editor

PLOS One
